# Leaching & electrochemical purification of mixed industrial LFP- & NMC type lithium-ion battery waste

**DOI:** 10.1039/d6ra04652e

**Published:** 2026-08-18

**Authors:** Johannes J. M. M. van de Ven, Maria-Alexandra Anghel, Patrick J. Teeuwisse, Yongxiang Yang, Shoshan T. Abrahami

**Affiliations:** a Delft University of Technology, Department of Materials Science and Engineering Mekelweg 2 2628 CD Delft The Netherlands S.T.Abrahami@tudelft.nl

## Abstract

The use of Li-ion batteries for electricity on the go and electrification of transport is rapidly increasing, resulting in rising pressure on critical raw materials (CRMs) such as Li, Co and Mn. However, commonly applied recycling routes have a large environmental impact, as extensive use of chemicals such as H_2_SO_4_ and H_2_O_2_ is required to achieve high dissolution rates. In addition, battery chemistries such as LFP (LiFePO_4_) have low intrinsic value, thereby limiting their recycling incentives. This research presents a recycling route for industrially pre-treated black mass (BM) from spent NMC (LiNi_*x*_Co_*y*_Mn_*z*_O_2_) and LFP batteries, combining a synergistic leaching step under mild acidic conditions (1 mol L^−1^ H_2_SO_4_) with subsequent removal of Fe, Al and Cu through a novel process that combines precipitation with electrochemical purification. Unlike conventional processes, the proposed simultaneous leaching resulted in the complete dissolution of Li, Co, Ni, Mn, and Fe without external reducing agents using industrially representative LiB-waste. A subsequent precipitation step, followed by electrodeposition of Cu and electrochemical oxidation of Fe^2+^ to Fe^3+^ using custom designed electrodes, was investigated at pH 2, 3, and 4. The best results were obtained by performing the electrochemical purification at pH 4. Overall removal efficiencies of 49% Al, 93% Fe and 73% Cu were achieved, of which Fe was mostly recovered as FePO_4_, while Ni, Co, and Mn co-precipitates with impurity levels as low as 0.1–0.6 wt% were obtained at pH > 8, leaving only Li in solution. This work presents a significant step towards more eco-friendly LiB-recycling, demonstrating the feasibility of a versatile, electrochemical purification approach.

## Abstract 2

A novel approach of simultaneous LFP- and NMC- type black mass leaching, followed by an electrochemical purification process, was applied to recycle Ni, Co, Mn, and Fe from mixed industrial Li-ion battery waste.

## Graphical abstract

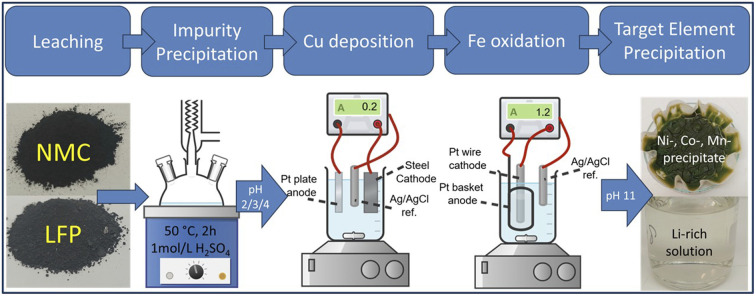

## 1. Introduction

Lithium-ion batteries (LiBs) are a crucial technology for electricity storage and play an essential role in the energy transition, with applications ranging from stationary energy storage to electric vehicles (EVs) and portable electronic devices (PEDs).^1–4^ These batteries rely on materials classified by the EU as critical raw materials (CRMs), such as Li, Co, and Mn, as well as the strategic raw materials (SRMs) Cu and Ni.^1,5,6^ However, while the demand for LiBs is expected to rise significantly in the coming years,^3,4,7^ the geopolitical supply risks associated with these elements are growing due to geographic concentration of primary production and growing international competition for these resources.^6,8^ Therefore, recycling will be an essential contributor to meet future demand, while also preventing landfilling or stockpiling of LiBs.^9^

Among the wide variety of techniques available for the recycling of LiBs, hydrometallurgy enables the recovery of a broad range of materials with high purities alongside limited energy intensity, making it the preferred route for future recycling.^10–13^ Hydrometallurgical recycling of LiBs typically requires an extensive pre-treatment that starts by shredding the batteries, producing a powder known as black mass (BM) that contains the majority of desired elements, including Li, Ni, Co, and Mn.^14^ Afterwards, the black mass is leached, typically in an acidic environment, to extract the metals into the liquid phase, called the *pregnant leach solution* (PLS).^15–18^ Researchers have found mineral acids such as HCl, H_2_SO_4_ and HNO_3_ to be particularly effective for this purpose, with studies reporting more than 99% dissolution of Li, Ni, Co, and Mn.^19–22^

Although promising results are reported with acid leaching, most studies emphasize the need to provide reducing conditions to dissolve Ni, Co, and Mn present in the cathode active materials (CAMs).^23–26^ This is because the average oxidation state of these elements in the solid CAMs is (III), while they are most soluble in state (II).^25–27^ Therefore, most hydrometallurgical routes rely on additional reducing agents, most commonly H_2_O_2_, Na_2_S_2_O_5_, or ascorbic acid, to enable complete dissolution of Ni, Co, and Mn.^23,28–34^ However, this is often accompanied by increased dissolution of impurities that are present in BM, including Fe, Al, and Cu. Moreover, the use of additional reagents increases the environmental footprint of the recycling process.^35,36^ Consequently, recent studies have shown that Fe(ii) from spent LiFePO_4_ (LFP) batteries can serve as an alternative to these reducing agents, which can avoid the need for additional reducing agents and strong acidic solutions.^24,37–42^

After leaching, Li, Ni, Co, and Mn must be effectively separated from Fe, Al, and Cu to create high grade battery precursors.^43^ A technique frequently applied in hydrometallurgy, and particularly in LiB-recycling, is selective precipitation.^15,18,44,45^ For example, Kang *et al.*^46^ used a solution containing 4 mol L^−1^ NaOH and 50 wt% CaCO_3_ to precipitate over 99% of Fe, Al, and Cu as hydroxides at pH 6.5. However, this purification step also resulted in a significant loss of Li, Ni, Co, and Mn (2%, 19%, 7%, and 15%, respectively). Chernyaev *et al.*^47^ demonstrated that carrying out Fe and Al precipitation at lower pH values of 3.5 for hydroxides and 3 for phosphates can minimize the coprecipitation of these valuable metals in a synthetic LiB PLS. In a later study, Zou *et al.*^48^ showed that 97.8% Fe removal is possible at a low pH of 2 in the presence of phosphates from simultaneous leaching of industrial NMC (LiNi_*x*_Co_*y*_Mn_*z*_O_2_) with pristine LFP, provided that iron is present as Fe^3+^, rather than the soluble Fe^2+^. A higher pH value of 4.5 allowed the removal of Fe together with Al and Cu but also induced substantial coprecipitation of Li, Ni, Co, and Mn. These results show that separation of Fe, Al, and Cu from Li, Ni, Co, and Mn by precipitation could only be achieved when, apart from a base to adjust pH, additional reagents such as phosphates or carbonates are used, since processes without such reagents reported major loss of the target elements.

In our previous study^24^ on simultaneous leaching of LFP and NMC, Fe removal efficiency was found to be lower when industrially pre-treated BM was used, which is a result of variations in need for reducing agents by industrial NMC BM, resulting in a PLS that contains both Fe^2+^ and Fe^3+^ ions. While Fe^3+^ can be readily removed at lower pH, any remaining Fe^2+^ remains soluble until higher pH, potentially coprecipitating with Ni, Co, and Mn products that are formed at a later stage of the recycling process.^49^ Given the high complexity of industrial BM which has a variable need for reducing agents as a result of varying oxidation states of Ni, Co, and Mn, and hence a resulting PLS with a fluctuating Fe^2+^ to Fe^3+^ ratio, a controlled and flexible processing step to oxidize any remaining Fe^2+^ is necessary before efficient removal can be achieved. While oxidation by air or pure oxygen is thermodynamically feasible, the reaction in acidic media is sluggish^50,51^ and therefore alternative methods need to be explored.

For both Cu and Fe, electrochemical methods offer a potentially more environmentally friendly approach for purification,^52^ although they have not yet been applied in the context of LiBs. After all, electrowinning of Cu has been extensively demonstrated in literature and in industry, both in primary production, as well as for the recycling of e-waste and other Cu-containing waste streams.^53^ Similarly, electrochemical methods for Fe removal have been demonstrated in other contexts, such as by Venkatesan *et al.*^54^ for recycling of NdFeB magnets. After leaching the magnets in HCl, an electrochemical setup with a large anode and small cathode was used to oxidize Fe from the NdFeB alloy to Fe^3+^, enabling its removal as FeCl_3_. These examples suggest that electrochemical removal of Cu and Fe could be a promising approach for a sustainable purification method, as it aligns with circular hydrometallurgical principles.^52^

In this study, we investigate the combined use of electrochemical and precipitation-based purification approaches and assess their application for industrially relevant conditions. We begin with optimizing the simultaneous leaching procedure for LFP and NMC^24^ using industrially pre-treated black mass (BM). This approach allows to replace H_2_O_2_ by Fe^2+^ from LFP dissolution as reducing agent. This leads to the observations that Fe^2+^ is not always completely oxidized to Fe^3+^ during leaching and that the use of Fe^2+^ as reducing agent promotes Cu dissolution. Hence, we subsequentially study the electrochemical oxidation of excess Fe^2+^ to Fe^3+^ and the electro-deposition of Cu, utilizing custom made anodes and cathodes with different size ratios to optimize for either Fe-oxidation or Cu-reduction. Its effectiveness is compared for three different solutions: a synthetic FeSO_4_-solution, a PLS from industrial LFP and a PLS from industrial LFP and NMC, allowing assessment of the process efficiency as a function of PLS complexity. We then determine the optimal pH of the electrochemical processes by comparing the composition of the purified PLS with and without prior electrochemical purification. Finally, the dissolved Ni, Co, and Mn that remained in solution are precipitated by incremental increases of the solution pH, leaving Li in solution. A comparison of the Ni, Co and Mn precipitates quality with- and without a prior electrochemical purification shows an improvement of 2.6% to 0.3% impurity content, hence showing the feasibility of the developed process.

## 2. Materials & methods

### 2.1. Battery materials

The battery materials used in this research were obtained from industrial partners and produced by dry mechanical pre-treatment under modified atmosphere followed by size classification, without additional heat treatment. Applied unit operations include shredding, sieving, magnetic separation, and grinding. Three different materials were used in this study. The NMC BM was used directly as received from the supplier. The LFP BM was also used as received in the first leaching experiment, but for the remainder of the experiments it was used after an additional size classification step that was performed in our lab (500 µm sieve retention) to decrease the amount of graphite, Al and Cu in this BM. Therefore, the as-received LiFePO_4_-type black mass is referred to as LFP BM and the sieved version of this black mass is referred to as LFPs BM. The composition of all BMs shown in Table 1. It should be noted that BM composition may change slightly over time due to the reactivity of its components. Therefore, these compounds were regularly analysed. The table represents the composition of the BMs at the beginning of the experiments. The standard deviations of the BM-characterizations, ranging from ±0.01–0.27 wt%, can be found in Table S1 in the SI.

**Table 1:** Composition (wt%) of all BMs used in this study as determined by aqua regia digestion and ICP-OES analysis. Other contains oxygen, graphite, and organic impurities

Material name	Li	Co	Ni	Mn	Al	Fe	Cu	PO_4_^3−^	Other	Note
NMC BM	3.8	6.7	16.8	6.5	0.5	0.9	0.9	—	63.9	BM containing NMC CAM
LFP BM	2.1	—	—	—	2.5	16.9	3	31.8	43.6	BM containing LFP CAM before sieving
LFPs BM	1.8	—	—	—	1.6	17.7	1.8	33.9	43.2	BM containing LFP CAM after sieving

### 2.2. Characterization techniques

The elemental compositions of the BM samples, leaching solutions, and precipitates were determined by inductively coupled plasma-optical emission spectroscopy (ICP-OES) using a Spectro Arcos-EOP device with a Modified Lichte nebulizer and mini cyclonic spray chamber. Calibration curves were made using 1000 mg L^−1^ ICP standards from each corresponding element. From the BM, 1 g of each sample was digested in 100 mL aqua regia, a combination of 25 mL HNO_3_ (65%, VWR chemicals) and 75 mL HCl (37%, Merck). The digestion was carried out in a triple necked round bottom flask with a reflux cooler, for 5 h at 70 °C. After this, the residue was separated through a Whatmann 595 ½ filter. The filtrate was diluted to 1 L in a volumetric flask and a sample of 0.5 mL from this solution was submitted to ICP-OES analysis. Each digestion was carried out at least three times. Over the course of the experiments, this characterization was repeated to ensure that changes in BM composition due to oxidation over time were accounted for.

The ferrozine method was used to determine the Fe^2+^/Fe^3+^ ratio in aqueous solutions.^55^ A solution containing ferrozine (4.9 g L^−1^, monosodium salt hydrate, 97%, Sigma-Aldrich) was used to form a Fe-complex measurable with UltraViolet-Visible spectrophotometry (UV-Vis). Using a Shimadzu UV-2600 apparatus, the absorption at 562 nm was measured, after which a reducing solution of 1.4 mol L^−1^ hydroxylamine hydrochloride (99%, Sigma-Aldrich) and 2 mol L^−1^ HCl (37%, Merck) was added. After a 10 min reaction, a pH buffer containing 10 mol L^−1^ CH_3_COONH_4_ (97%, Alfa Aesar), which was adjusted to pH 9.5 with NH_3_-solution (25%, VWR chemicals), was added and the absorption at 562 nm was measured again. Comparing the difference in absorption before and after reduction provides the Fe^2+^/Fe^3+^ ratio.

For characterization of BM, Cu deposits, and precipitates, identification of chemical phases was required, for which X-ray diffraction (XRD) analysis was conducted. A Bruker D8 Advance diffractometer with Bragg–Brentano geometry and Lynxeye-XE-T position sensitive detector was used for the analysis, with Cu Kα radiation. The 2*θ* range was 5–110°, with a step size of 0.03 and 1 s per step. Data analysis was done *via* the Bruker software DiffracSuite.EVA vs 7.2.

### 2.3. H_2_SO_4_ leaching of LFP & NMC

Leaching was performed to optimize the dissolution of Li, Ni, Co, and Mn from NMC in presence of LFP, as well as to acquire a solution suited for further purification. The laboratory scale leaching setup consisted of a 1 L round bottom flask, which was placed in a heating mantle on a stirring and heating plate. A temperature probe was inserted through one of the necks for temperature control and a reflux cooler was attached to another neck. Stirring was achieved with a magnetic stir bar. The lixiviant had a total volume of 500 mL and contained H_2_SO_4_ (95.0–97.0%, Sigma-Aldrich), diluted in Milli-Q water. The process was carried out for 2 h total with 600 rpm stirring. After this reaction, the slurry was filtered in two steps, once through a vacuum filtration with a Whatmann 113 filter (90 mm diameter, 30 µm pore size), followed by membrane filtration through a Cytiva ME 25 membrane filter with 0.45 µm pore size. The residue was dried for 8 h at 55 °C, and the PLS was collected for further study.

**Fig. 1:**
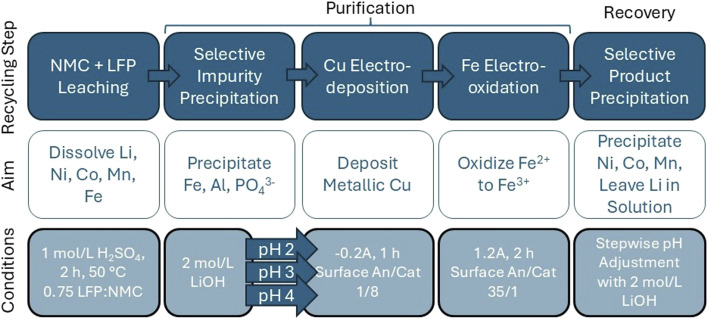
Overview of recycling approach. Cu-deposition, Fe-oxidation, and subsequent selective precipitation were carried out three times, at pH 2, 3, and 4.

In the first leaching experiment (Fig. 1), NMC BM was leached in 0.63 mol L^−1^ H_2_SO_4_ at 50 °C. The solid-to-liquid (S/L) ratio of the NMC BM is 38.4 g L^−1^, which was based on the amount of Ni, Co and Mn that was present in the solutions of van de Ven *et al.*.^56^ After 30 min, when a portion of NMC was already dissolved, LFP BM was added in a molar equivalent based on the ratio of the sum of (Ni + Co + Mn) in the NMC BM compared to Fe in the LFP BM. This strategy lowers the initial peak S/L ratio during the leaching process, while theoretically resulting in a total S/L of 104 g L^−1^. Then, at a total time of 90 min, 1 vol% of H_2_O_2_-solution (30% Sigma-Aldrich) is added to ensure complete leaching, after which it is allowed to react for 30 more mins. This brings the total reaction time to 2 h.

In the following leaching experiments (Fig. 3), 1 mol L^−1^ H_2_SO_4_ was used at 50 °C together with the sieved LFPs BM. A lower molar ratio of 0.75 LFP : NMC was used due to the presence of Cu and Al which, together with Fe, exhibit catalytic reducing effect.^24^ The total S/L was also increased to more closely resemble industrial conditions, resulting in 57.6 g L^−1^ NMC BM and 67.7 g L^−1^ LFPs BM. The LFPs BM was added in three equal steps at 30, 60, and 90 minutes of leaching, after which it was allowed to react for 30 more minutes, resulting in 2 h total. No H_2_O_2_ was added.

The leaching progress over time was studied by sampling 1.5 mL at each 15 minutes interval, after which the slurry was filtered with a syringe filter (Fisherbrand PTFE 0.45 µm). Of this filtrate, 0.1 mL was submitted to ICP-OES analysis.

To assess the effectiveness of the leaching process, the leaching efficiency (*η*_L_) of the elements of interest was calculated according to eqn (1). In this formula, *m*^x^_f_ represents the initial mass of element x in the feed, which was determined by the analysis of the BM as described in Section 2.2. *m*^x^_PLS_ represents the mass of element x present in the PLS after leaching. This was determined by ICP-OES analysis of the PLS. Over three leaching experiments, the standard deviations of the leaching efficiencies of Li, Co, Ni, Mn, Al, Fe and Cu were found to be 5.1%, 4.6%, 0.5%, 4.0%, 3.3%, 1.4%, and 4.5%, respectively, showing good repeatability of the experiments.1
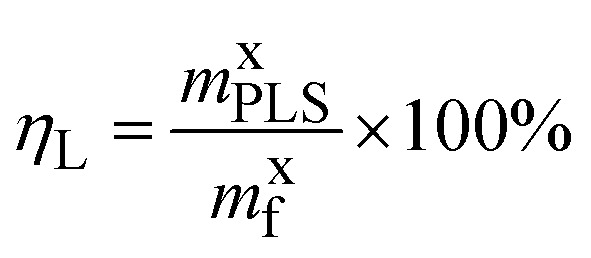


### 2.4. Selective precipitation

After leaching or electrochemical treatment, selective precipitation by pH adjustment was applied. This was done using a 2 mol L^−1^ LiOH solution (monohydrate, ≥99%, Fischer Scientific). After the first leaching experiment without electrochemical treatment, to account for concentration changes in the PLS caused by dilution, the solution was weighed before and after LiOH addition. This was found to cause overestimation of the remaining element in solution. In all other experiments, including after electrochemical treatment, the dilution was accounted for by measuring the initial volume each step with a volumetric cylinder, while the LiOH solution was added using a Metrohm 775 dosimat. After this change, correction for dilution no longer resulted in an overestimation of the concentrations except for Al and Cu impurities. At each desired pH, the solution suspension was filtered with a vacuum filtration pump using a Büchner funnel and Whatmann 542 filter paper (90 mm diameter, 2.7 µm pore size). The filtrate (0.1 mL) was diluted in 3 wt% HNO_3_ and submitted to ICP-OES analysis. The remaining filtrate was weighed again, after which the following pH adjustment was done.

After these steps were carried out for all applied pH increments, the collected precipitates were dried (55 °C, 8 h), and weighed. Then, 0.1 g of each precipitate sample was dissolved in 5 wt% HNO_3_, sometimes assisted by the addition of H_2_O_2_ (35% solution, Thermo Scientific) in a 100 mL volumetric flask. Of this solution, 0.5 mL was submitted to ICP-analysis.

### 2.5. Electrochemical techniques

For all electrochemical purification experiments, a Parstat 4000 (Ametek, UK) potentiostat was used, controlled with the Versastudio software. For the electro-oxidation, a Pt basket of 5 * 11 cm was used as anode (working electrode), while a Pt wire was used as cathode (counter electrode). This results in an anode-to-cathode surface ratio of roughly 35 : 1. Electro-oxidation was carried out using the chronopotentiometry method, at 1.2 A resulting in a current density of 21.8 mA cm^−2^. The setup is visualised in Fig. S1 in the SI. To ensure the reliability and reproducibility of electro oxidation, the first test was conducted on a FeSO_4_-solution which contained 0.18 mol L^−1^ Fe, similar to the PLS from simultaneous leaching (0.173 mol L^−1^ Fe). Then, it was conducted on an LFP PLS obtained by leaching the LFPs BM with the same conditions as in the combined leaching, but in absence of NMC BM, resulting in 0.2 mol L^−1^ Fe.

In the Cu deposition experiments, a 0.5 cm wide Pt plate anode was used as counter electrode, while a steel plate of 4 cm wide submerged for 4 cm was used as cathode (working electrode). The anode-to-cathode-ratio was 1 : 8, and a current of 0.2 A (12.5 mA cm^−2^) was applied on the cathode through chronopotentiometry. The setup is visualised in Fig. S2 in the SI. Both experiments made use of a triple electrode setup with a saturated Ag/AgCl electrode as a reference electrode. All reported potentials in this work are relative to the standard hydrogen electrode (SHE). The experiments were performed in a beaker with 250 mL of solution, magnetically stirred at 400 rpm and room temperature. During electrochemical purification, samples of 1.5 mL were taken at regular intervals and filtered through a syringe filter (Fisherbrand PTFE 0.45 µm). A sample of 0.1 mL from this solution was submitted to ICP-OES analysis, while 0.03 mL was submitted to UV-Vis analysis. The overall experimental approach, in which the electrochemical purification processes were conducted on the PLS, which was either raised to pH 2, 3, or 4, is presented in Fig. 1.

## 3. Results & discussion

### 3.1. Simultaneous leaching of NMC- & LFP black mass followed by pH-controlled precipitation

**Fig. 2:**
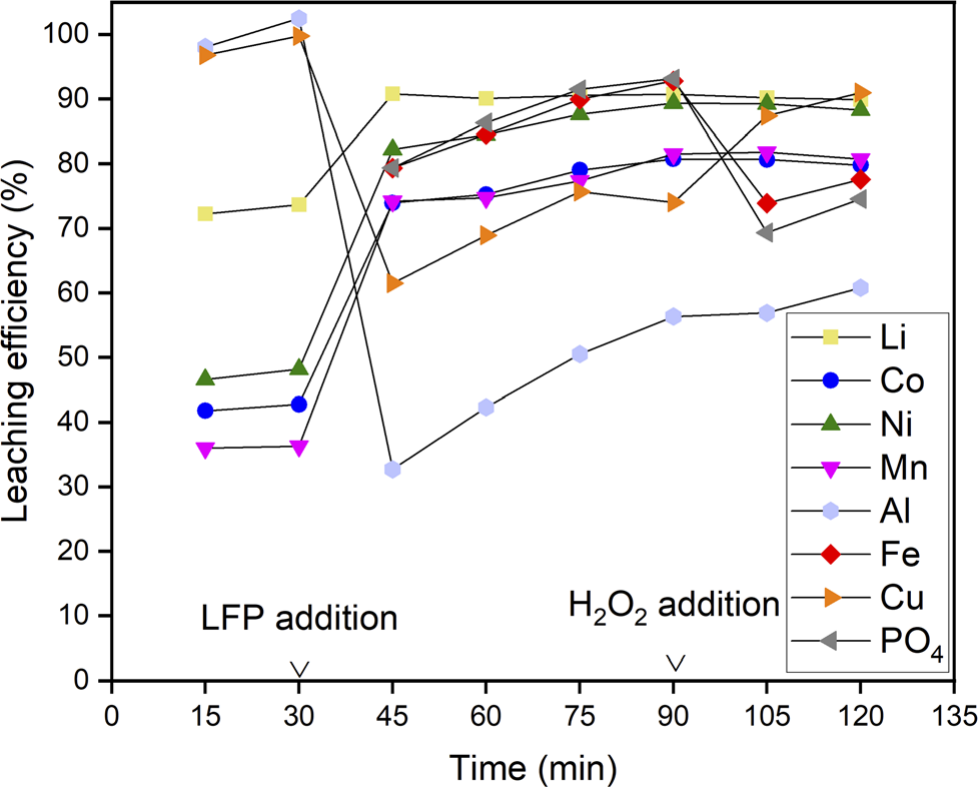
Dependence of the leaching efficiency (0.63 mol L^−1^ H_2_SO_4_, 50 °C) of Li, Co, Ni, Mn, Cu, Fe, Al, and PO_4_^3−^ from NMC BM (38.4 g L^−1^) on time, as well as the presence of LFP BM (65.5 g L^−1^) and H_2_O_2_ (1 vol%).

The effect of Fe^2+^ on NMC leaching has been demonstrated in our previous study.^24^ However, this was conducted using a manually liberated LFP cathode material. To bring the method one step closer to practical conditions, industrially pre-treated samples were used in the present study. The results of leaching efficiency as function of time for different elements/species are illustrated in Fig. 2.

These results show that the initial dissolution of NMC BM in 0.63 mol L^−1^ H_2_SO_4_ is low (around 40% Ni, Co, and Mn and 74% for Li), while the low amounts of Al and Cu impurities that are present in the NMC BM completely dissolve. It has to be noted that the 102% leaching efficiency for Al is not possible in practice, and that this is a result of the heterogeneous nature of industrially pre-treated black mass, especially regarding the content of impurities. The leaching behaviour is well-known in the literature and arises because Ni, Co and Mn in a higher oxidation state must first be reduced to their divalent state to dissolve.^23,27,29,34,57^ Only under such conditions, the dissolution of NMC BM follows the reaction presented in eqn (2), resulting in efficient dissolution. Addition of LFP BM after 30 minutes results in the presence of Fe^2+^ in solution, according to eqn (3), which can act as a reducing agent according to eqn (4). This leads to a rapid increase in leaching efficiencies, as the dissolution of LFP and the subsequent reaction of Fe^2+^ are very fast.^39,58^ This is followed by a more gradual increase in the leaching efficiencies of Ni, Co, and Mn, which follow the gradual increase of Cu and Al in solution. The slower reactions of these species with Fe^3+^ contribute to the regeneration of Fe^2+^, which subsequently promotes the reduction and dissolution of Ni, Co and Mn.^24,39,58^ These three elements reach 89%, 81%, and 81% after a total reaction time of 90 min (of which 60 min with LFP BM), respectively, while also 91% of Li is dissolved, even though its feed concentration almost doubles upon LFP BM addition. The leaching efficiencies of Cu and Al are lowered (74% Cu and 56% Al) due to their increased concentration after LFP BM addition (from 0.2 to 1.2 g L^−1^ for Cu, and from 0.2 to 1.8 g L^−1^ for Al).

The addition of H_2_O_2_ at the 90 min mark increases the dissolution of Cu and Al to 91% and 61%, respectively, as a result of their oxidation. Moreover, about 20% of the Fe and PO_4_^3−^ in solution is removed upon H_2_O_2_ addition, which can be attributed to the oxidation of the Fe^2+^ by H_2_O_2_. Although this reaction involving free radicals is complex and unpredictable, the overarching reactions are shown in eqn (5) and (6).^59^ Due to the low solubility of Fe^3+^ in the presence of PO_4_^3−^, FePO_4_ precipitates according to eqn (7).^24^ This behaviour was not observed in our previous research, as H_2_O_2_ reacts with unoxidized Fe^2+^ in presence of reduced Ni, Co and Mn, as well as with unreduced Ni, Co, and Mn in presence of oxidized Fe^3+^. Since both unoxidized Fe and unreduced Ni, Co, and Mn are present in the current study, and H_2_O_2_ is a stronger oxidizing agent than a reducing agent,^60^ it favours Fe oxidation (eqn (5) and (6)) rather than the reduction of Ni, Co, and Mn. Moreover, the ions Fe^2+^, Mn^2+^, Ni^2+^, and Co^2+^, have standard reduction potentials of +0.77 V, +1.23 V, +1.59 V and +1.92 V, respectively. Hence, Fe^2+^ has the strongest tendency to get oxidized by H_2_O_2_, and any oxidized Mn-, Ni- or Co-products will react with Fe^2+^ nevertheless, forming Fe^3+^. As a result, the leaching of Ni, Co, and Mn is unaltered by H_2_O_2_ addition.

2 3LiNi_1/3_Mn_1/3_Co_1/3_O_2 (s)_ + 12H_(aq)_^+^ + 3e^−^ → 3Li_(aq)_^+^ + Ni_(aq)_^2+^ + Mn_(aq)_^2+^ + Co_(aq)_^2+^ + 6H_2_O_(l)_

3 LiFePO_4(s)_ + 2H_(aq)_^+^ → Li_(aq)_^+^ + Fe_(aq)_^2+^ + H_2_PO_4 (aq)_^−^

4 Fe_(aq)_^2+^ → Fe_(aq)_^3+^ + e^−^

5
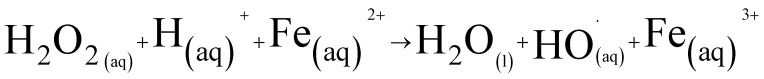
6
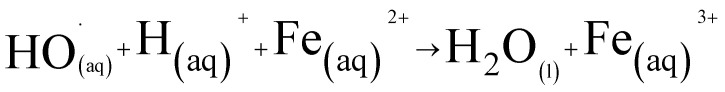

7 Fe_(aq)_^3+^ + H_2_PO_4 (aq)_^−^ → FePO_4 (s)_ + 2H_(aq)_^+^

It is important that the introduced Fe, Al, and Cu should not contaminate any Ni, Co, and Mn compounds aimed to be recovered downstream. Selective precipitation is a widely used purification technique in hydrometallurgy, is straight forward, can directly result in battery precursors, and is therefore attractive in LiB recycling. To study the applicability of this technique after simultaneous leaching of LFP and NMC, the PLS from this experiment was subjected to precipitation by incremental pH adjustments. This was done by adding a 2 mol L^−1^ LiOH solution to the PLS until a desired pH, after which the solution was filtered and analysed on remaining elements.

**Fig. 3:**
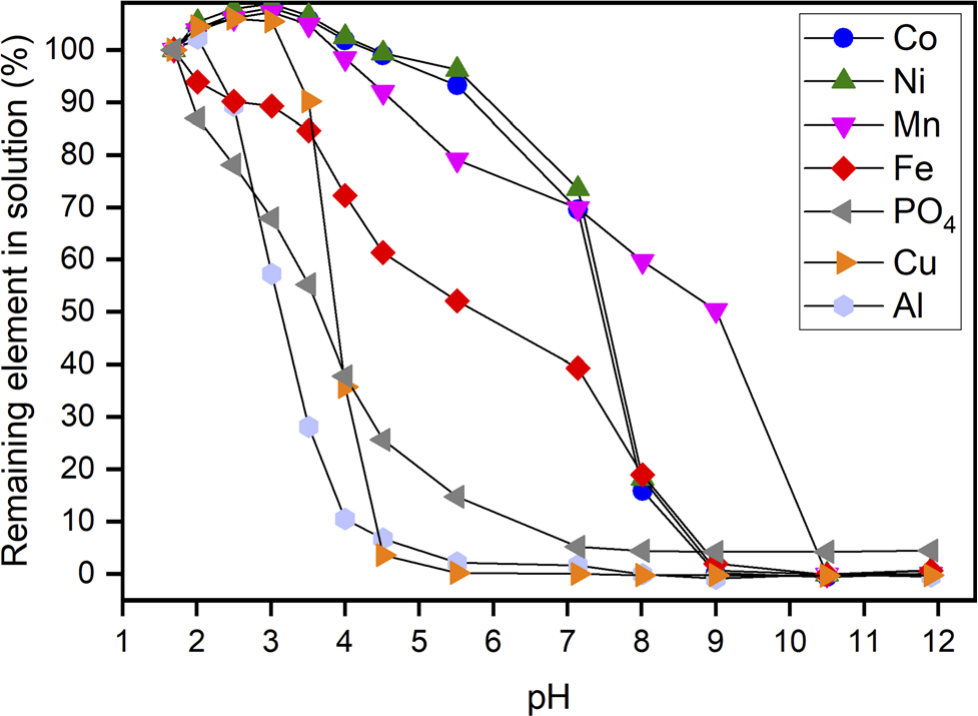
The remaining elemental concentration compared to the PLS at different pH values, as achieved by stepwise precipitation after simultaneous leaching of NMC BM and LFP BM.

Fig. 3 shows the percentages of elements remaining in the PLS *vs.* pH, after correcting for dilution. Increasing the pH from 1.7, the final pH of the PLS, to pH 4.0 causes most of the Al to precipitate from the solution. This can be seen by its steep decrease in concentration, resulting in 10.5% of the initial concentration remaining at pH 4.0. Cu exhibits a similar trend, with only 3.5% remaining at pH 4.5. At this pH, roughly 97% of the Ni, Co, and Mn remain in solution, indicating a 3% loss of these valuable elements. A further pH increase shows that Co and Ni mainly precipitate between pH 5.5 and pH 9.0, as the concentrations decline steeply within this pH range, while Mn precipitates above pH 9.0. In contrast, Fe does not show a sharp decrease within a narrow pH window but exhibits a gradual decrease over the entire pH range, resulting in significant Fe contamination in all precipitates. This is confirmed by analysis of the precipitates which shows a minimum Fe content of 4 wt% across all pH steps. PO_4_^3−^ shows the largest decrease of all elements between pH 1.7 and 2.5, resulting in 22% being precipitated. Subsequentially, it keeps declining alongside Al, Cu, and Fe, respectively. Especially after pH 4.5, the trend is nearly identical to that of Fe. The results for Ni, Co, and Mn are similar to what Wang *et al.*^61^ found when precipitating Li, Ni, Co, Mn, Cu, Al, and Fe from a PLS of industrial NMC BM, through pH increase with NaOH. However, Cu and Al precipitate between pH 2 and 5 in the current research, presumably due to phosphate presence lowering the solubility of these two impurities, while in the study of Wang *et al.* they precipitate between pH 3 and 6. Moreover, the Fe in the study of Wang *et al.* precipitates entirely by pH 4.5, which is a result of its low feed concentration (0.3 wt% in the BM) compared to the current research, in which it is present for 16.9% in the LFP BM. As Fe coprecipitates in the entire pH range, potentially resulting in low quality Ni, Co, and Mn products, further process optimization to minimize Fe-contamination is necessary.

**Fig. 4:**
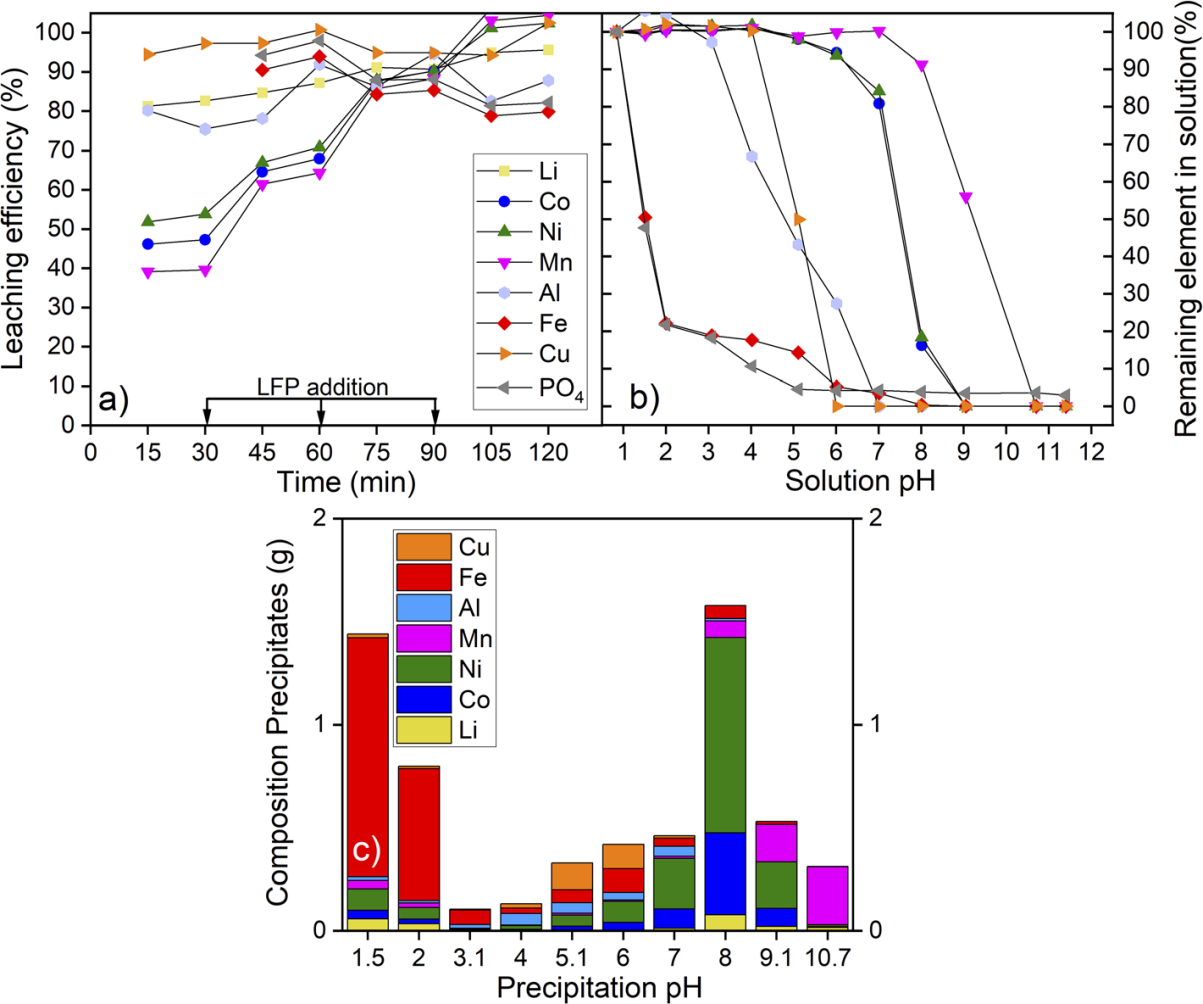
(a) Leaching efficiency of Li, Ni, Co, Mn, Fe, Al, Cu, and PO_4_^3−^ from NMC BM (57.6 g L^−1^) as a function of time, with stepwise addition of LFPs BM (65.2 g L^−1^). (b) Remaining elemental concentration compared to the PLS at different pH values, as achieved by stepwise precipitation after simultaneous leaching of NMC BM and LFPs BM. (c) Composition of resulting precipitates.

It is important to note that the concentration of all elements, with the exception of Al, Fe, and PO_4_^3−^, seems to increase at the beginning of the precipitation step. This can mainly be attributed to the nature of the correction by dilution by the LiOH-solution, which is based on the increase in solution mass. However, experimental uncertainties that arise during filtration as well as different density of the PLS and LiOH-solution make complete correction impossible. Hence, in the following experiment (Fig. 4), dilutions were measured by volume additions, resulting in a significantly more accurate correction Another important point to be made is that the precipitates that are formed are largely amorphous, apart from the presence of Li_2_SO_4_ and a minor amount of FePO_4_, as shown by XRD-analysis. As examples, the diffractograms of the precipitates formed at pH 2.0, 4.0, and 8.0 are shown in the SI (Fig. S3). Amorphous, or homogeneous, precipitation causes impurities to absorb on the surface of the formed precipitates, hence removing them from the solution.^62^ This is why the concentration of Ni, Co and Mn lowers already from pH 4 onwards, as these are lost in the homogeneous precipitates of Fe, Al and Cu.

The reason for the wide precipitation range of Fe is most likely the result of the two oxidation states in which it is present. While Fe^3+^ has low solubility above pH 2, Fe^2+^ remains soluble at higher pH from 5 even up to 11 as a result of ion pair formation with hydroxides, resulting in soluble FeOH^+^.^49,63,64^ Hence, Fe^2+^ contaminates any precipitates that are formed at pH 3 and above. Moreover, presence of metals such as Cu and Al in industrially pre-treated BM leads to additional redox reactions with Fe^3+^ during leaching, as described in literature.^24,65^ This essentially increases the reducing capacity of LFP BM beyond the calculated amount based on TMs (Ni, Co, Mn) and Fe molar ratios. Another point of interest is the PO_4_^3−^ precipitating faster than Fe. Together with the analysis of the formed precipitates, which indicate the presence of Al, Cu, and PO_4_^3−^, this suggests precipitation of AlPO_4_ and Cu_3_(PO_4_)_2_.

To avoid an excess of Fe^2+^ in the PLS, improvements were made to the reagents and leaching procedure. Firstly, size classification (sieving), a commonly practiced method in industry, lowers the amount of graphite, Al, and Cu in the as-received LFP BM. Secondly, the molar ratio of LFPs BM to NMC BM was lowered from 1 to 0.75 to account for the “excess” reducing capability of Cu and Al. Thirdly, the LFPs BM was added in three steps of 0.25 molar ratio at 30, 60, and 90 min of leaching, as this can avoid high initial S/L while being a relatively simple process control parameter. Fourthly, raising the acid concentration to 1 mol L^−1^ H_2_SO_4_ accommodated a higher total S/L of 122.8 g L^−1^, which is closer to industrially relevant conditions compared to the initial leaching procedure. As a result, leaching could be completed without the need for H_2_O_2_, as is shown in the leaching results over time in Fig. 4a. The subsequent precipitation and corresponding precipitate compositions are shown in Fig. 4b and c, respectively.

The leaching efficiencies of the target elements at the start of the experiment are slightly higher (6–7%) compared to the ones observed in the previous conditions (Fig. 2), with an average of 46% Co, Ni and Mn dissolved and 83% of Li. This is most likely due to the higher acid concentration (1 mol L^−1^ instead of 0.63 mol L^−1^), which compensates for the higher S/L of the NMC BM (57.6 *vs.* 38.4 g L^−1^).

At 45 minutes (15 min after the first dose of LFPs BM), the dissolution of Ni, Co, and Mn rises to 68% on average, while for Li it rises to 87% despite its increased feed concentration from 1.6 to 1.9 g L^−1^. Similar increases of approx. 18% are seen after the second and third dose of LFPs BM addition, which are all a result of Fe(ii) from LFP readily dissolving (eqn (3)), promoting the reduction of Ni, Co, and Mn from the NMC BM. Finally, after 2 h of total leaching, Ni, Co, and Mn are completely dissolved, with the efficiency of Li reaching 95%. Around 95% of Cu and 88% Al dissolve throughout the leaching process according to the mechanism described earlier in this research. The leaching efficiencies of Fe and PO_4_^3−^ are always high, starting at 92%, 15 min after LFPs BM addition (45 min mark) and dropping to 80% at the end of the leaching experiment, as a result of the increasing S/L of LFPs BM.

With the leaching process further optimized, it is necessary to evaluate its influence on the subsequential purification by pH adjustment. The composition of the remaining PLS at different pH values can be seen in Fig. 4b, while the composition of the resulting precipitates is shown in Fig. 4c. It is important to note here that the initial increase in Al concentration is a result of experimental uncertainties related to dilution by the LiOH-solution, and is more pronounced than for the other elements due to the low absolute Al concentration. Compared to the previous precipitation results (Fig. 3), there is a significant improvement in the separation of Fe, Al, and Cu from Li, Ni, Co, and Mn. Upon reaching pH 2.0, 78% of the Fe is removed, as the Fe^2+^ from the dissolved LFPs BM was consumed in the reduction of Ni, Co, and Mn, to a greater extent as a result of leaching parameter optimization and feed material sieving, resulting in a high amount of poorly soluble Fe^3+^ in the PLS. At pH 5.1, only 10% of Fe and PO_4_^3−^ is left in solution, while the Cu and Al remain for 50% and 43%, respectively. As a result, the precipitates formed at pH 1.5, 2.0 and 3.1 mainly consist of Fe and PO_4_^3−^, whereas the precipitates at pH 4.0 and 5.1 also contain Al and Cu. However, Ni and Co start to precipitate from pH 5.1 onwards, as can be seen by their steeper decrease in concentration beyond this point. As the higher degree of FePO_4_ precipitation between pH 1.5 and 3.1 leaves less PO_4_^3−^ available to precipitate with Al and Cu, there is a significant overlap in the precipitation of these impurities with Ni and Co, which is also confirmed by precipitate analysis in Fig. 4c. It shows that the precipitates formed at pH 6.0 and pH 7.0 are a mixture of Ni, Co, Fe, Al and Cu. Moreover, even the precipitate formed at pH 8.0, which contains the largest amount of Ni and Co, is contaminated with Fe. The remaining Ni and Co precipitate at pH 9.1, whereas the Mn precipitates between pH 8 and 10.7.

Due to the higher removal of Fe by merit of the more carefully controlled leaching process, these precipitates are significantly less contaminated with impurities. However, large amounts of contamination in the precipitates formed between pH 5.1 and pH 8.0 show that solely adjusting leaching does not result in sufficient separation of Cu, Al, and Fe from Li, Ni, Co, and Mn, and that there is a need for further purification steps, starting with the oxidation and removal of Fe.

### 3.2. Electrochemical oxidation of Fe^2+^ to Fe^3+^ after leaching

**Fig. 5:**
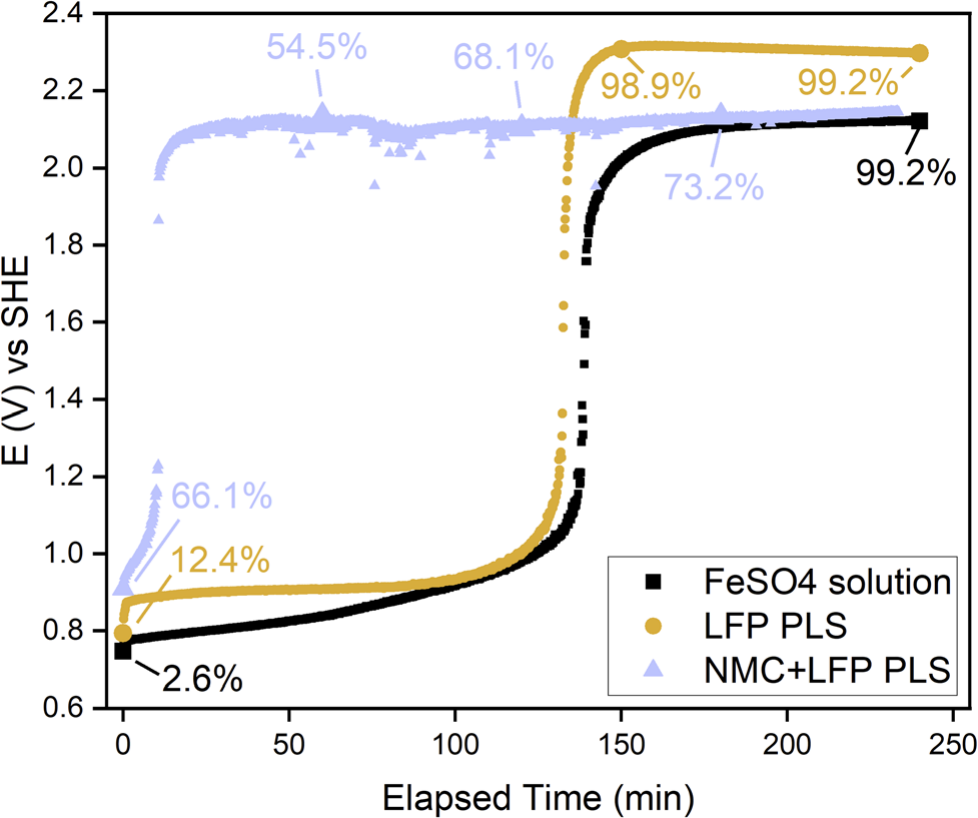
Evolution of the anode potential over time during chronopotentiometry (1.2 A) performed on a FeSO_4_-solution (0.18 mol L^−1^), LFPs BM-PLS, and NMC BM + LFPs BM-PLS. Percentages of Fe^3+^ compared to total Fe concentration as calculated from UV-vis measurements using the ferrozine method are indicated.

To investigate the electrochemical oxidation of Fe as a function of PLS complexity, we start by oxidizing the Fe in a simple, synthetic, solution made of FeSO_4_ in 1 mol L^−1^ H_2_SO_4_. This is performed in an electrochemical cell with a large anode to cathode ratio, thereby promoting the Fe oxidation at the anode, according to eqn (4), with hydrogen gas formation at the cathode, resulting in the overall cell reaction in eqn (8). After this initial test, we repeated the process on a PLS made from leaching of LFPs BM alone. This simplified PLS is used to test the process on industrial BM without interference from additional metal ions originating from NMC BM. Next, the method is repeated on the optimized PLS resulting from simultaneous leaching of LFPs BM and NMC BM (Fig. 4a). A comparison of the measured potential in the three described solutions over time is shown in Fig. 5, together with the Fe^3+^*vs.* total Fe percentage at selected measurement points.

8 2H_(aq)_^+^ + 2Fe_(aq)_^2+^ → H_2 (g)_ + 2Fe_(aq)_^3+^ (Δ*E* = 0.77 V)

The cell potential of the FeSO_4_-solution starts at *E* < 0.77 V *vs.* SHE, which is the standard potential of *E*^0^ = 0.77 V for the oxidation of Fe^2+^ to Fe^3+^ but very soon reaches 0.78 V. Over the course of 125 min, the potential gradually increases to 1.05 V, after which it sharply rises to 2.1 V, where it stabilizes around that value (±0.1 V). A similar curve is observed for the PLS containing only LFPs BM, starting at a slightly higher potential of 0.9 V for the first 120 min, before it suddenly increases to 2.3 V, a few minutes earlier than in the FeSO_4_-solution. The curve for the PLS from simultaneous leaching (NMC BM + LFPs BM) also starts around 0.9 V, but it sharply increases after 10 min, rising to an anode potential of 2.1 V, where it stabilizes over the remaining 230 min. The shape of these curves reflects the decrease in Fe^2+^ concentration, accompanied by an increase in Fe^3+^ concentration, in accordance with the Nernst equation in eqn (9), where *R* is the ideal gas constant, *T* the temperature, *n* the number of electrons, and *F* the Faraday constant. It has to be noted that in eqn (9), the activity coefficients of Fe^3+^ and Fe^2+^ are approximated by their concentrations. The dependence of the Fe oxidation on the cell potential is visualized in the SI, Fig. S6.9
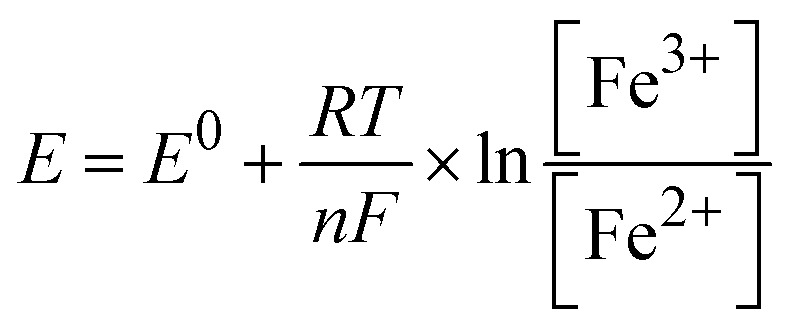


Once the concentration of Fe^2+^ has dropped significantly, the solution no longer has sufficient Fe^2+^ to sustain the redox process and a sharp increase in anode potential toward 2 V marks the transition from Fe^2+^ oxidation to water electrolysis. At this point, the oxygen evolution reaction (OER) becomes the dominant anodic process, which is also indicated by gas formation at the anode. This trend is also confirmed by Fe^3+^/Fe^2+^ ratio analysis using UV-Vis. Before electro-oxidation, only 2.4% of the Fe in FeSO_4_-solution was in the Fe^3+^ state. This increased to 99.2% after 240 min of electro-oxidation. In the simple LFPs BM PLS, 12.4% of the Fe was initially present as Fe^3+^, which possibly oxidized during the processing of the BM, leaching or due to battery aging. This number increased to 98.9% after 150 min, and 99.1% after 240 min of electro-oxidation. The shapes of the potential curves for the FeSO_4_-solution and the LFPs BM PLS are both similar to the curves in the study of Venkatesan *et al.*,^54^ who also investigated a relatively simple solution containing predominantly Fe^2+^ and Nd^3+^ in HCl. Hence, it is clear that electro-oxidation is an effective method to oxidize Fe^2+^ in solutions containing predominantly Fe, and monitoring the anode potential provides a reliable indication for the oxidation progress.

The distinct curve of the electro-oxidation in the PLS containing LFP- and NMC BM can be attributed to multiple factors. Firstly, the amount of Fe that was already in the oxidized state, Fe^3+^, directly after leaching was significantly higher (66.1%). This is due to redox reaction with the Ni, Co and Mn from the NMC BM, which are reduced by the Fe^2+^ during leaching. Secondly, after 180 min the oxidation to Fe^3+^ increased by a mere 7% (to 73.2%). This was accompanied by the deposition of a red-brown coloured deposit at the cathode, which XRD-analysis revealed to contain Cu and Cu_2_O (Fig. S7). Cu^2+^ in solution makes its reduction on the cathode (*E*^0^ = 0.34 V, eqn (21), Table 2) thermodynamically more favoured compared to the hydrogen evolution under the applied conditions. Moreover, the deposits also increase the surface area of the cathode which, together with the increasing Fe^3+^ concentration in solution now leads to its reduction back to Fe^2+^ becoming more favoured (*E*^0^ = 0.77 V), leading to reaction “loop” and low overall oxidation efficiency. Thirdly, any deposited Cu can react with Fe^3+^ through the same reaction as during leaching, essentially resulting in a zero net operation. Fourthly, apart from Cu, reduction of other elements at the cathode such as Al, Mn, Fe, Co, and Ni according to eqn (11) to (15) are plausible at such high cell potentials. ICP-OES analysis of a cathode deposit in a later experiment confirms the presence of all of these elements. This suggests that these can interfere with efficient Fe oxidation since their metallic deposits can react with Fe^3+^, similarly to the leaching reactions, producing Fe^2+^ again. Lastly, side reactions at the anode, such as the oxidation of Co^2+^ to Co^3+^ (eqn (29), +1.92 V) and Mn^2+^ to Mn^3+^ (eqn (27), +1.51 V), can lead to further efficiency losses. These reactions compete with Fe^2+^ oxidation, since the potential reaching 2.1 V provides sufficient driving force. While these trivalent cations can still facilitate Fe^2+^ oxidation by reacting with it in solution, the formation of insoluble oxides such as NiO_2_ (eqn (28)), CoO_2_ (eqn (26)), or MnO_2_ (eqn (25)) is detrimental to the process. Analysis of the anode deposit in a later experiment confirms the presence of Mn, Ni, and Co alongside Fe, confirming the formation of insoluble oxides.

**Table 2:** Reduction half reactions relevant for the LFPs + NMC PLS

Reduction half reaction	*E* ^0^ (V)^60^	Eqn no.
Li^+^ + e^−^ → Li	−3.04	(10)
Al^3+^ + 3e^−^ → Al	−1.676	(11)
Mn^2+^ + 2e^−^ → Mn	−1.18	(12)
Fe^2+^ + 2e^−^ → Fe	−0.44	(13)
Co^2+^ + 2e^−^ → Co	−0.282	(14)
Ni^2+^ + 2e^−^ → Ni	−0.257	(15)
H_3_PO_4_ + 2H^+^ + 2e^−^ → H_3_PO_3_ + H_2_O	−0.276	(16)
Fe^3+^ + 3e^−^ → Fe	−0.044	(17)
2H^+^ + 2e^−^ → H_2_	0	(18)
HSO_4_^−^ + 3H^+^ + 2e^−^ → H_2_SO_3_ + H_2_O	+0.158	(19)
Cu^2+^ + e^−^ → Cu^+^	+0.159	(20)
Cu^2+^ + 2e^−^ → Cu	+0.340	(21)
Cu^+^ + e^−^ → Cu	+0.520	(22)
Fe^3+^ + e^−^ → Fe^2+^	+0.77	(23)
O_2_ + 4H^+^ + 4e^−^ → 2H_2_O	+1.23	(24)
MnO_2_ + 4H^+^ + 2e^−^ → Mn^2+^ + 2H_2_O	+1.23	(25)
CoO_2_ + 4H^+^ + e^−^ → Co^3+^ + 2H_2_O	+1.4	(26)
Mn^3+^ + e^−^ → Mn^2+^	+1.51	(27)
NiO_2_ + 4H^+^ + 2e^−^ → Ni^2+^ + 2H_2_O	+1.59	(28)
Co^3+^ + e^−^ → Co^2+^	+1.92	(29)

### 3.3. Electrodeposition of Cu & subsequent electro-oxidation of Fe

While the presence of Cu^2+^ interferes with the complete oxidation of Fe^2+^, it also presents an interesting opportunity to remove Cu electrochemically and recover it in its metallic state. To maximize Cu and Fe removal after simultaneous leaching, we investigated a purification strategy that includes pH adjustment to 2, 3 or 4, followed by a two-step electrochemical purification process consisting of Cu electrodeposition and Fe oxidation (Fig. 1 in Section 2). Table 3 shows the concentration of all elements in the solution at different steps of the process, including after the pH increase, Cu deposition, and Fe oxidation.

**Table 3:** Concentration of elements in solution at the end of different stages in the recycling process, measured by ICP-OES. Decreases in concentration which are not due to dilution (volume increase is indicated as “*V* × *x*”) are highlighted in green, together with the corresponding percentages between brackets (relative to the initial PLS). For comparison, the solution after leaching industrial BM + spent LFP and precipitating to pH 3 from van de Ven *et al.* is also reported

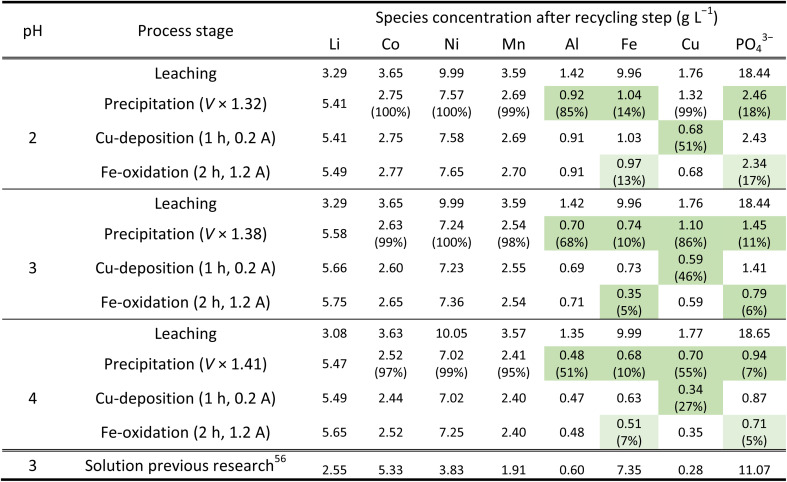

Comparing the concentration of Fe and PO_4_^3−^ after the first precipitation step, we can conclude that about 86% of the present Fe precipitates as FePO_4_ at pH 2, similarly to the results shown in Fig. 3. Precipitation increases at higher pH, reaching 89.7% at pH 3 and 90.4% at pH 4. Apart from dilution, Cu is not affected by adjusting the solution to pH 2, while 15% of Al precipitates at this point. However, 32% of Al and 14% of the Cu precipitate at pH 3, while roughly 47% of both Cu and Al are removed by adjusting the solution to pH 4. Important to note as well is that raising the pH to 2 causes no significant loss of Ni, Co, and Mn due to coprecipitation, while raising to pH 3 or 4 only results in roughly 1% and 2.5% loss of these elements on average, respectively.

During the second purification step, Cu deposition results in 49% of Cu removal at pH 2, while at pH 3 and 4, 53% and 73% are removed, respectively. For all three pH values, the concentrations of Ni, Co, and Mn do not change in the process. These findings indicate that Cu deposition is reasonably efficient at all tested pH values, which is a result of the low anode-to-cathode surface ratio (1 : 8), promoting Cu deposition at the larger cathode, and limiting the premature oxidation of Fe^2+^ to Fe^3+^ at the smaller anode, in favour of the oxygen evolution reaction (eqn (24)). Also, since the pH is increased, any Fe that does oxidize to Fe^3+^ precipitates, avoiding interference with Cu deposition and resulting in high selectivity for Cu. This is apparent from the fact that the concentration of all other species during Cu deposition is nearly unaffected, and that qualitative ICP-OES analysis of the deposits on the cathode shows few impurities at pH 2 and 3. However, the deposit at pH 4 also contains a significant amount of Co and Ni. Qualitative analysis shows a ratio of 1 : 2 for Co : Cu, and 1 : 4 for Ni : Cu. When looking at eqn (14) and (15) in Table 2, this can be easily explained. The cathode potential (*vs.* SHE) during deposition starts at −1.7 V, and eventually reaches −1.4 V, as can be seen in Fig. S8 in the SI. This is sufficient to reduce Ni^2+^ and Co^2+^ to their metallic form (*E*^0^ = −0.26 and −0.28, resp.).

During subsequent Fe^2+^ oxidation, little changes in the elemental concentrations are measured, with a 1–5% decrease of Fe and PO_4_^3−^. The system at pH 2 only shows a 1% decrease in Fe and PO_4_^3−^ concentration, since this pH is too low for any oxidized Fe to precipitate. The same process at pH 3 also only shows a change in Fe and PO_4_^3−^ concentration, from 10.5% to 5.5% on average, lowering the concentration from 0.73 to 0.35 g L^−1^ and 1.41 to 0.79 g L^−1^, respectively. Surprisingly, the oxidation at pH 4 only decreases Fe by 2% or 0.12 g L^−1^. This could be a result of the lower PO_4_^3−^ concentration at the start of the oxidation due to prior AlPO_4_ precipitation. Also, the pH after the Fe oxidation decreased from 4 to 2.4, resulting in higher solubility of Fe^3+^, thereby lowering the efficiency of its removal. The Ni and Co concentrations are unaffected throughout all experiments, but the Mn concentration lowers by 1% during the electro-oxidation at pH 4. ICP-OES analysis of the deposit on the anode shows the presence of Mn.

When comparing the resulting solution obtained after electrochemical purification at pH 4 with the one obtained by van de Ven *et al.*^56^ through leaching of an industrial NMC BM with spent LFP and subsequent pH adjustment to 3.0, the effectiveness of the presented recycling approach is further established. Improved leaching conditions result in a higher amount of Ni, Co, and Mn in total, being 12.17 g L^−1^ (after 40% dilution by pH increase) rather than 11.07 g L^−1^, while Li numbers are hard to compare due to different pH adjustment media (LiOH *vs.* NaOH). Cu and Al concentrations are similar in the current research *versus* the results of our previous study, being 0.35 *vs.* 0.28 g L^−1^ Cu, and 0.48 *vs.* 0.60 g L^−1^ Al, both despite a much higher leaching efficiency in the current leaching method. Most importantly however, the Fe concentration in the current research is significantly lower, being 0.51 *vs.* 7.35 g L^−1^, as a result of electrochemical purification.

### 3.4. Selective precipitation of Ni, Co, & Mn after electrochemical purification

**Fig. 6:**
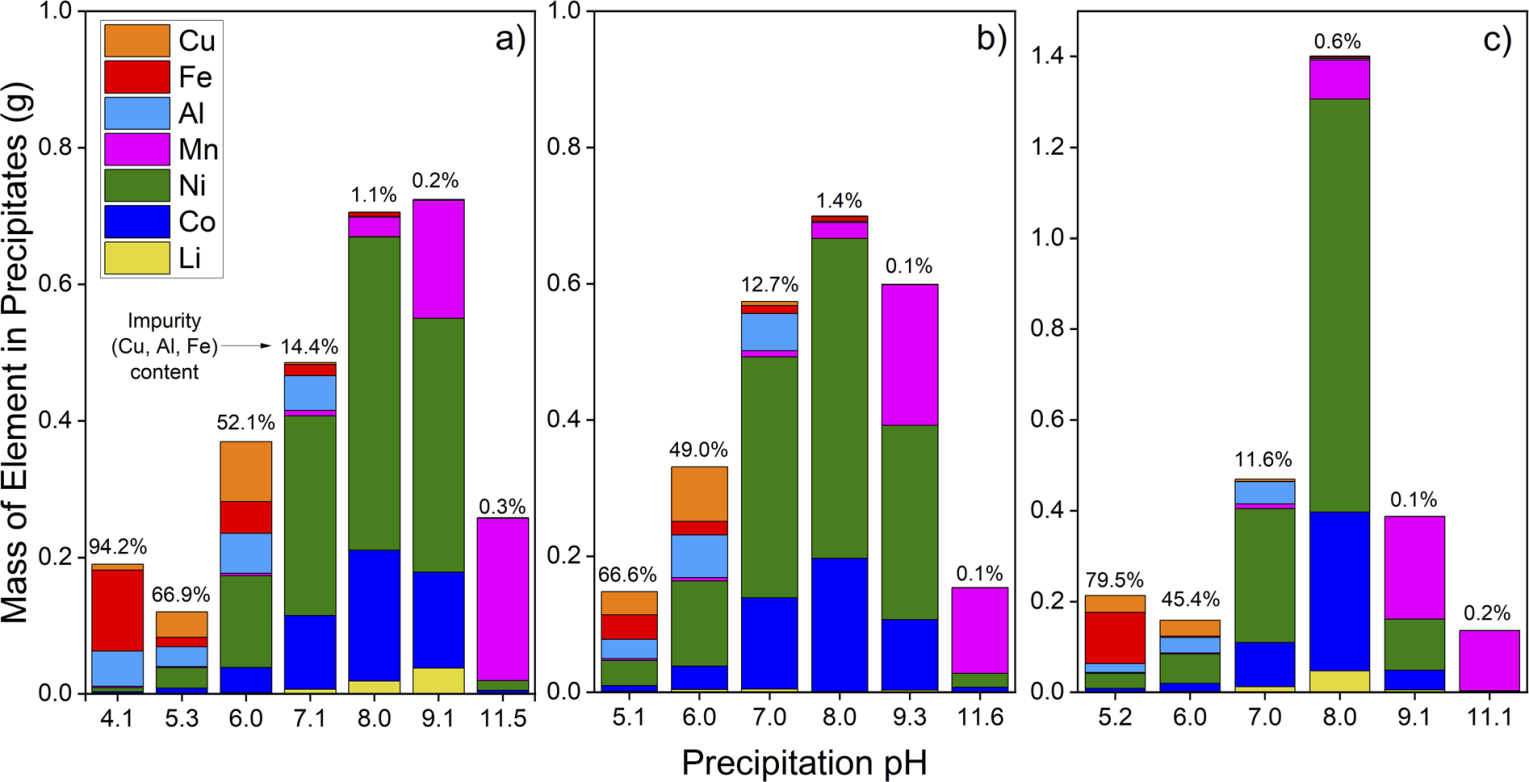
Composition of the precipitates formed through selective precipitation after electrochemical purification at pH 2 (a), pH 3 (b), and pH 4 (c). Above each bar, impurity content on metal basis according to eqn (30) is shown.

Besides the impurity removal efficiency, the composition and purity of the recovered target element compounds are of great importance in a hydrometallurgical process. In our case, the target elements Ni, Co, and Mn are recovered by a second precipitation through pH adjustment, which was conducted after electrochemical treatments at different pH values, analogous to the selective precipitation in Fig. 3, leaving Li in solution. To emphasise the purity of the resulting precipitates rather than the remaining elemental concentration over the pH increase, only the composition of the precipitates formed as a result this procedure are shown in Fig. 6. To compare how the purity of the precipitates is influenced by the pH at which electrochemical purification is conducted (2, 3 or 4), the impurity content of the precipitates on metal basis is calculated according to eqn (30). The mass components in this equation represent the mass of the corresponding element that is present in the precipitate obtained at pH *X*. These values are reported above the respective bar in Fig. 6.30
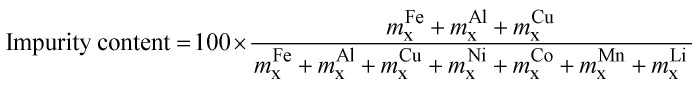


Fig. 6a shows the compositions of the precipitates formed after electrochemical purification at pH 2. The first precipitate at pH 4.1 mostly consists of Fe, Al and Cu impurities (94.2%) while the precipitate at pH 5.3 contains significantly less, being 66.9%. At this point, having such high impurity content is desirable since the selective precipitation is targeted at removing Fe, Al, and Cu, rather than recovering Ni, Co, and Mn. However, the precipitate at pH 5.3 containing 33.1% target elements indicates a significant loss. Further raising the pH to 6.0, at which the target elements Ni and Co start precipitating significantly, results in a precipitate containing 52.1% impurities, while the following precipitate at pH 7.1 mostly contains target elements and only 14.4% impurities. The precipitates at pH 8.0, 9.1, and 11.5 represent the main recoveries of the target elements, and merely consist of 1.1%, 0.2% and 0.3% impurities, respectively. This is already a significant improvement to the selective precipitation without prior electrochemical purification, as presented in Fig. 3c, which produced precipitates at pH 8.0, 9.1, and 10.7 containing 4.7%, 2.8%, and 0.5%, respectively. However, more improvement is left to be desired when comparing to precipitation studies such as the one from Kang *et al.*,^46^ who achieved 99% precipitation of Fe, Al, and Cu associated with a loss of 19% Ni, 7% Co, and 15% Mn, while the present results show 99% precipitation of Fe, Al, and Cu alongside a nearly 30% loss of Ni and Co.

In Fig. 6b, the precipitates after electrochemical purification at pH 3 are presented. The solids formed at pH 5.5 and 6.1 are similar to their counterparts from Fig. 6a, and contain much contamination by Fe, Al, and Cu, being 66.6% and 49.0%, respectively. The precipitate at pH 7.0 has a large amount of Ni and Co, but still contains 12.7% impurities of which most is Al, which is problematic regarding the high amount of Ni and Co that is hence lost. Beyond this point, the precipitates are of good quality, showing 1.4%, 0.1% and 0.1% impurities at pH 8.0, 9.3, and 11.6, respectively.

In Fig. 6c, the compositions of the precipitates acquired after electrochemical purification at pH 4 are shown. Here, the precipitate formed at pH 5.2 again shows the largest presence of Fe, Al, and Cu, totalling 79.5%. The solids formed at pH 6.0 and 7.0 show lower impurity contents of 45.4% and 11.6%, respectively. Similarly to the other two scenarios, this results in a large loss of target elements due to overlap between the target element and impurity coprecipitation. Further precipitates at pH 8.0, 9.1, and 11.1 merely contain 0.6%, 0.1%, and 0.2% impurities, respectively, showing the highest purity of the main target element precipitates.

Comparing the three sets of precipitates leads to the conclusion that electrochemical purification at higher pH leads to precipitates with fewer impurities. Although the metal basis purities at pH 8.0 for the experiments at pH 2, 3, and 4 are 96.3%, 98.5%, and 96.0% (not shown in graph), respectively, also looking at impurities, which make up 1.1%, 1.4%, and 0.6%, respectively, vouches for this trend. The precipitates around pH 9 also have lower impurity content in case of electrochemical purification at pH 2, 3, and 4 with values of 0.2%, 0.1%, and 0.1%. This again shows that selective precipitation after electrochemical treatment at pH 4 produces the purest precipitates, which is mostly a result of a more effective Cu deposition during this process. Conversely, at pH 5 or lower, high impurity content is desired, indicating good removal of Fe, Al, and Cu with minimal loss of target elements. The differences in impurity contents at pH 5 in Fig. 6a, b, and c (66.9%, 66.6%, and 79.5%) again indicate prior electrochemical purification at pH 4 being superior, since it results in the precipitate with the highest impurity content of 79.5%. Moreover, comparing the weighted average impurity content of the Ni, Co, and Mn precipitates (≥ pH 8) resulting from the purification at pH 4 (Fig. 6c) with the ones without electrochemical purification (Fig. 4c), which are 0.5% and 3.7%, respectively, confirms the effectiveness of the presented recycling process. However, the overall yields of Ni, Co, and Mn, calculated from the precipitates in Fig. 6c obtained above pH 8, are only 48%, 48% and 54%, respectively. These relatively low yields are primarily due to the fact that they are calculated only based on the fraction of precipitates fulfilling the high purity requirements (<0.6%), while precipitates obtained between pH 5 and 8 also contain the target elements, but with impurity levels exceeding these requirements (impurities between 11.6-79.5%). Comparing these values with the overall recycling efficiencies reported by Kang *et al.* (92% Co from BM using solvent extraction), Wang *et al.*^66^ (95–98% Ni, Co and Mn from BM using cementation and selective precipitation) or Lu *et al.*^67^ (98.4–99.7% Ni, Co and Mn from manually dismantled LiBs using co-precipitation), indicates that further improvements are needed. It is, however, possible to internally recycle intermediate products or streams, such as mixed precipitates with insufficient purity back to earlier leaching step to improve overall recovery and reduce losses, although improved separation selectivity is still desired. Further process optimization, particularly the separation of Fe, Al, and Cu below pH 5 and thereby increasing the precipitation yields of Ni, Co and Mn, is still necessary before the presented recycling approach can become feasible for upscaling.

## 4. Conclusions

This study developed an improved method to simultaneously leach LFP- and NMC- based BM and demonstrates its applicability to industrially pre-treated LiB-waste. It also combines electrochemical purification with recovery by precipitation in a novel hydrometallurgical recycling flowsheet. First, complete dissolution of Ni, Co, and Mn is achieved with the assistance of Fe^2+^, originating from LFP dissolution, as a reducing agent. Al and Cu impurities, originating from the current collectors, regenerate Fe^2+^ upon dissolution, increasing the reducing capability of the LFP BM, avoiding the need for external reducing agents. The optimized leaching parameters are 1 mol L^−1^ H_2_SO_4_, 125.3 g L^−1^ total LFPs BM and NMC BM (0.75 molar ratio), and a total leaching time of 2 h at 50 °C. Although Fe^3+^ can be readily removed from the PLS at pH 2, Fe^2+^ does not precipitate until pH 8, and consequently, contaminates the Ni, Co, and Mn precipitates. Electrochemical oxidation of Fe^2+^ using a large anode and small cathode (surface area ratio of 35 : 1) and a current density of 21.8 mA cm^−2^ (1.2 A) successfully oxidises Fe^2+^ to Fe^3+^, improving its separation from Ni, Co and Mn by impurity precipitation. This approach also offers a major processing advantage, since it enables *in situ* control of the Fe oxidation progress by monitoring the potential as described by the Nernst equation. This is a relevant feature for industrial recycling, which can apply different ratios of LFP : NMC in the leaching step. However, it was found that the enhanced dissolution of Cu hinders electrochemical oxidation of Fe^2+^, since Cu deposits on the cathode where it can react with Fe^3+^ to regenerate Fe^2+^ and Cu^2+^, drastically lowering the oxidation efficiency. This electrochemical reaction loop can be avoided by first increasing the pH of the PLS to precipitate the Fe^3+^ as FePO_4_. Subsequently, Cu is removed *via* electrodeposition at 0.2 A (12.5 mA cm^−2^), followed by the Fe^2+^ electro-oxidation. This sequence of processing steps was investigated as a function of solution pH (2, 3 or 4), with the optimal results obtained at pH 4, where 93% of the Fe, and 73% of the Cu were removed. Subsequent selective precipitation through incremental pH increase resulted in improved separation of the remaining Fe, Al, and Cu from the target elements. Electrochemical purification at pH 4 resulted in Ni, Co, and Mn precipitation products at pH > 8 containing little impurities (on average 0.5% *vs.* 3.7% without electrochemical purification), leaving only Li in solution. The overall recovery yield under these processing conditions were 48%, 48% and 54%, respectively for Ni, Co and Mn, and 68% for FePO_4_. Hence, while this work presents a significant step towards more eco-friendly LiB-recycling, further optimization is needed to improve Cu removal and address Al separation, which remained the primary contaminant in the Co, Ni, and Mn end products.

## Author contributions

Johannes J. M. M. van de Ven: conceptualization, methodology, investigation, formal analysis, validation, writing – original draft. Maria-Alexandra Anghel: investigation, validation. Patrick J. Teeuwisse: investigation, validation. Yongxiang Yang: writing – review and editing, supervision. Shoshan T. Abrahami: conceptualization, writing – review and editing, supervision, funding acquisition.

## Conflicts of interest

The authors declare that they have no known competing interests.

## Supplementary Material

RA-OLF-D6RA04652E-s001

## Data Availability

The datasets used and/or analysed during the current study are available at the 4TU Research data repository (DOI: 10.4121/58947ce2-c651-43cc-8e4d-760b2e60adfb). Supplementary information (SI): illustrations of experimental setups, XRD-analysis results of precipitates, and visualisations of chronopotentiometry data. See DOI: https://doi.org/10.1039/d6ra04652e.
