# Supplementary material for: Leaching & electrochemical purification of mixed industrial LFP- & NMC type lithium-ion battery waste
Source: RSC Adv. 2026 Aug 18;16(41):48513–26. doi: 10.1039/d6ra04652e (PMC13482751; doi:10.1039/d6ra04652e)
Supplement: Supplementary file 1 [file RA-016-D6RA04652E-s001.pdf]

This journal is © The Royal Society of Chemistry 2026

Supplementary Information: Leaching and Electrochemical Purification of Mixed Industrial LFP- and NMC type Lithium-ion Battery Waste

Black mass characterization

Table S.1: Standard deviation of the composition (wt.%) of all BMs used in this study as determined by aqua regia digestion and ICP-OES analysis. The standard deviation is characteristic of the digestion and ICP-measurement, as well as the heterogeneity of the BMs on one given moment. It does not represent any reactivity or compositional changes of the BMs over time.

Material Name	Li	Co	Ni	Mn	Al	Fe	Cu	PO_4^{3-}
NMC BM	0.06	0.08	0.22	0.08	0.01	0.01	0.01	-
LFP BM	0.05	-	-	-	0.05	0.24	0.08	0.27
LFPs BM	0.01	-	-	-	0.01	0.04	0.01	0.12

Electrochemical Setups

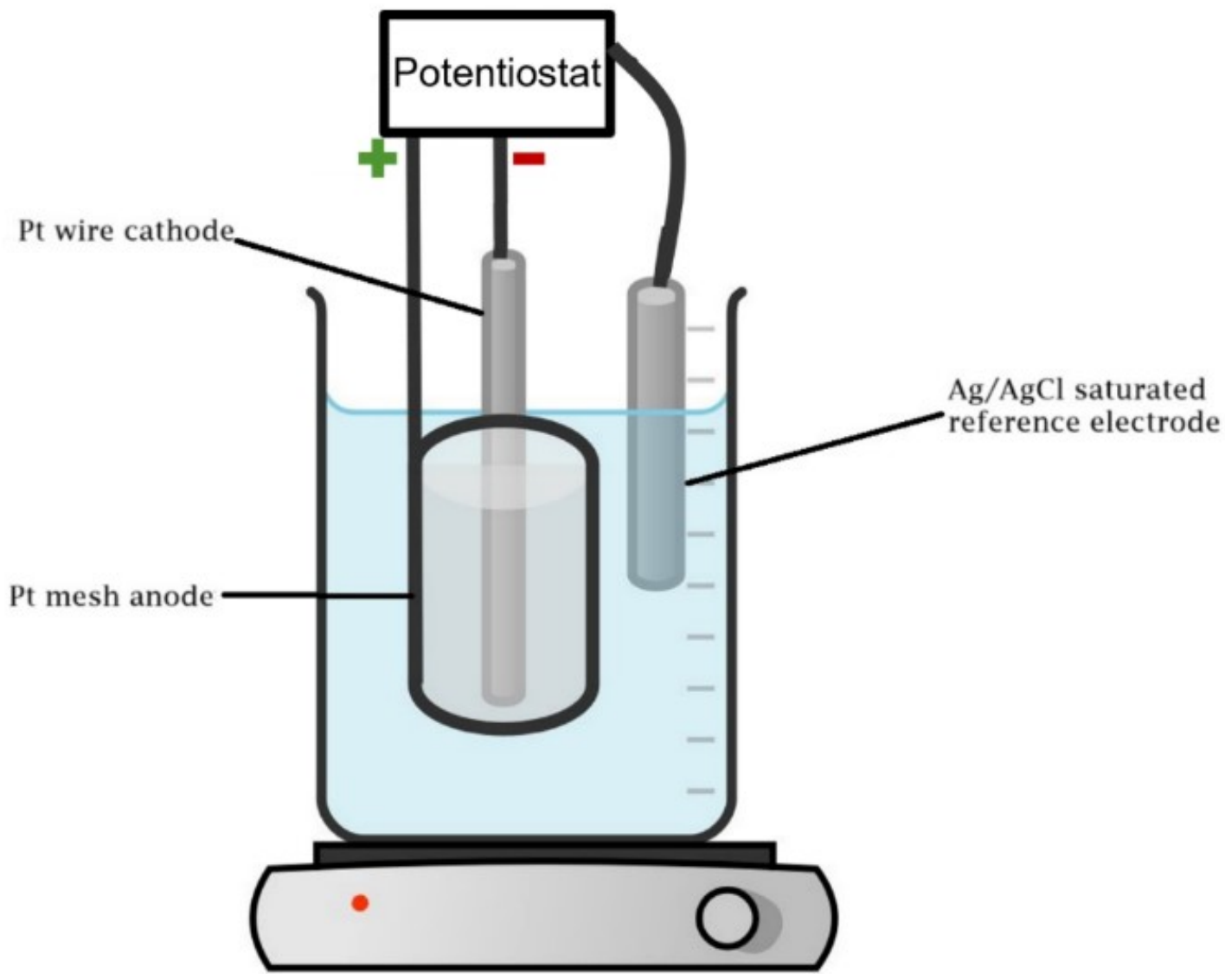

Figure S.1: Visualisation of the setup used for electrochemical oxidation of Fe^{2+} to Fe^{3+}.

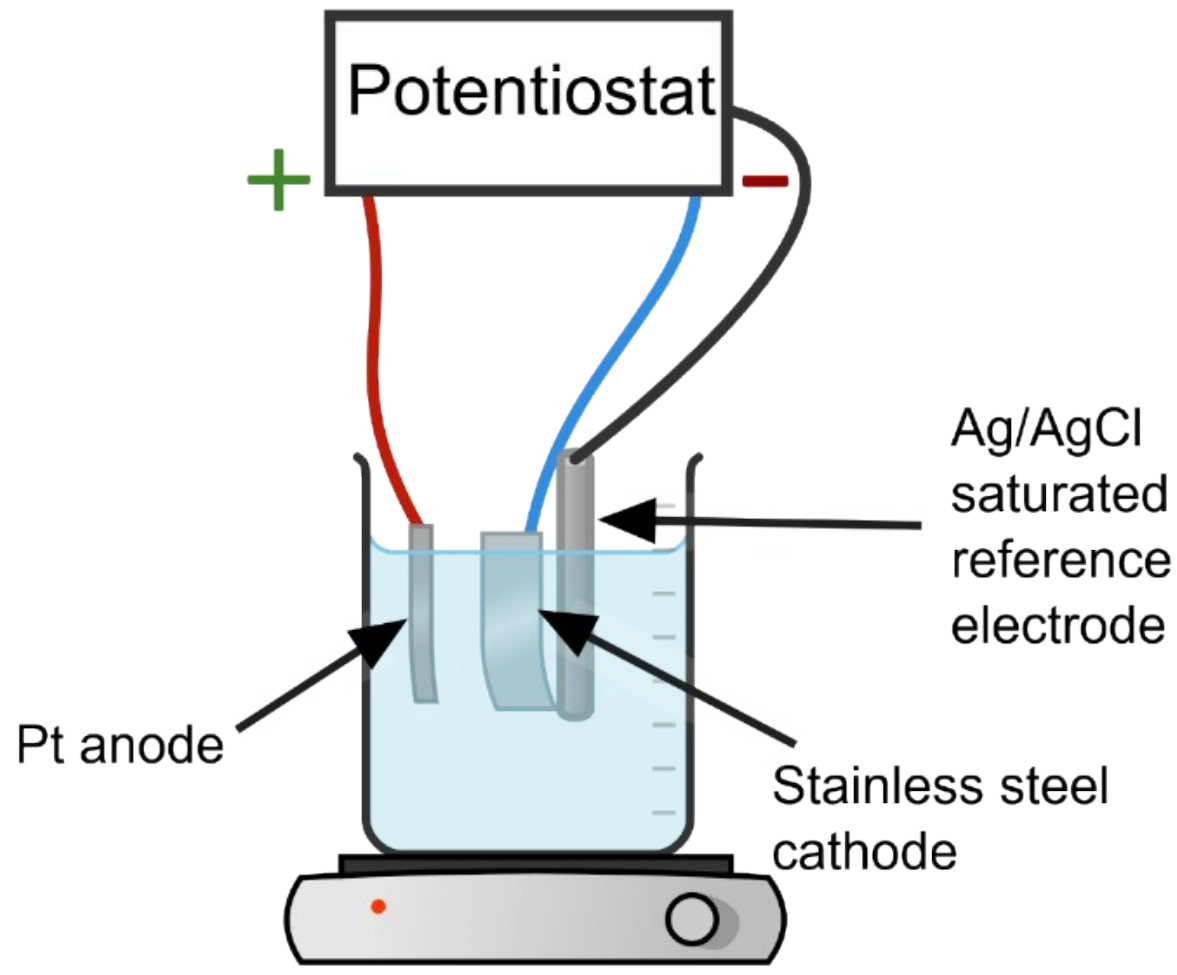

Figure S.2: Visualisation of the setup used electro-deposition of Cu

XRD-analysis Preciptitates

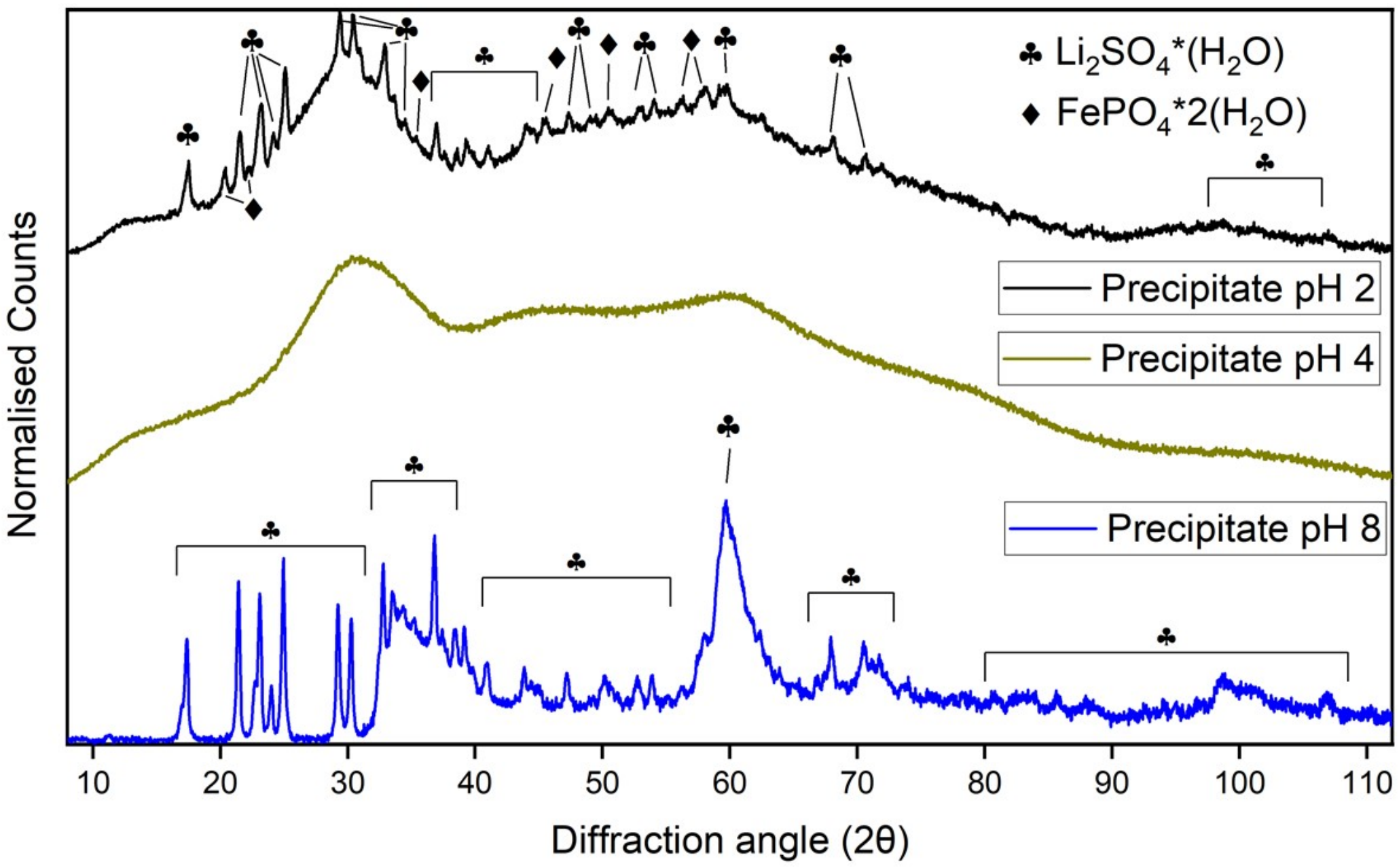

Figure S.3: XRD-diffractograms of the precipitates formed after leaching of LFP BM and NMC BMs.

In Figure S.3, it is shown that the precipitates presented in Figures 3 and 4 are predominantly amorphous, with minor amounts of $FePO_4$ and more substantial presence of Li_2SO_4 identified. For comparison, Fig S.4 and S.5 show the diffraction patterns of Li_2SO_4 and $FePO_4$, respectively.

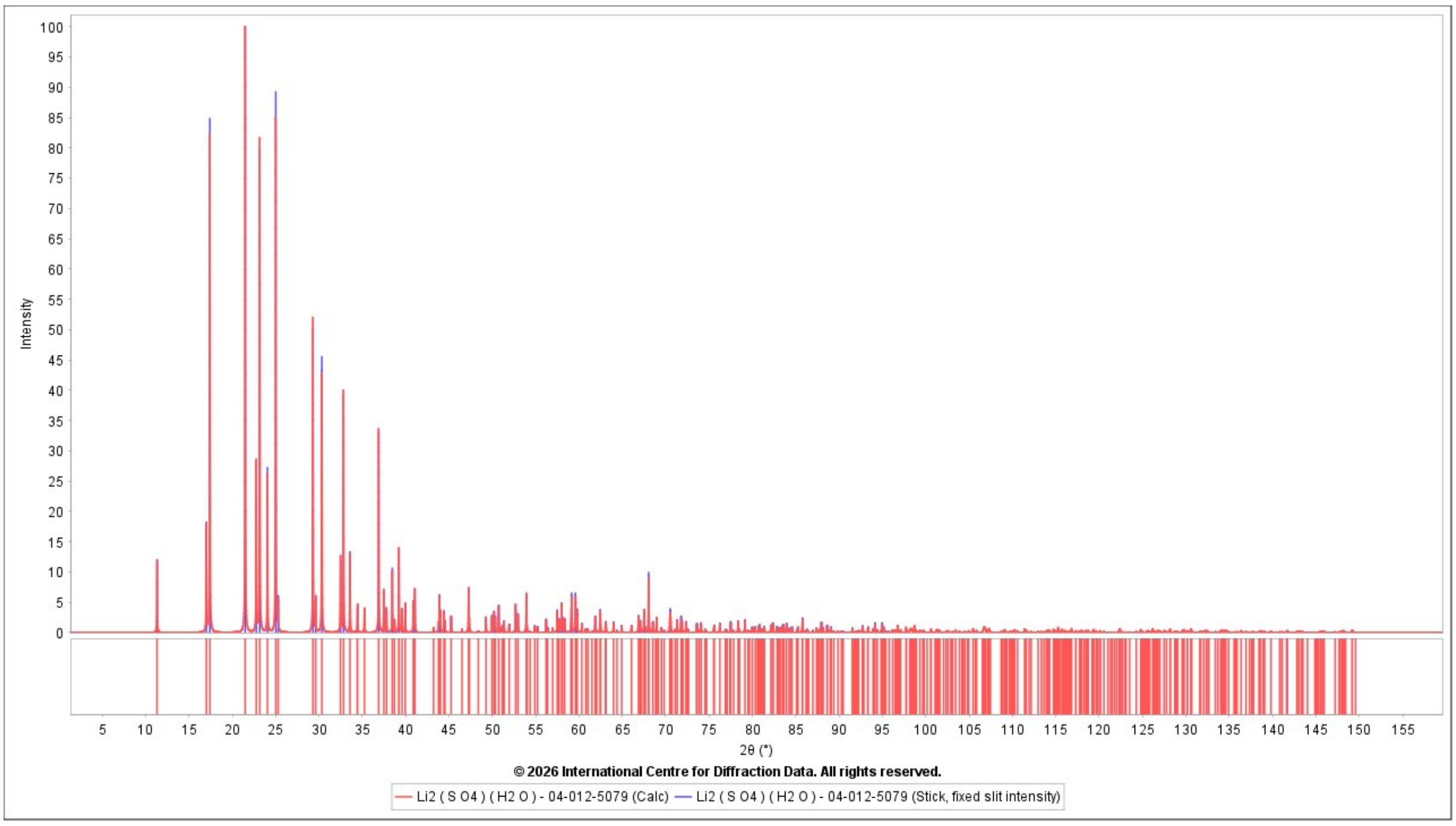

Figure S.4: Diffraction pattern of pure Li_2SO_4(H_2O)*

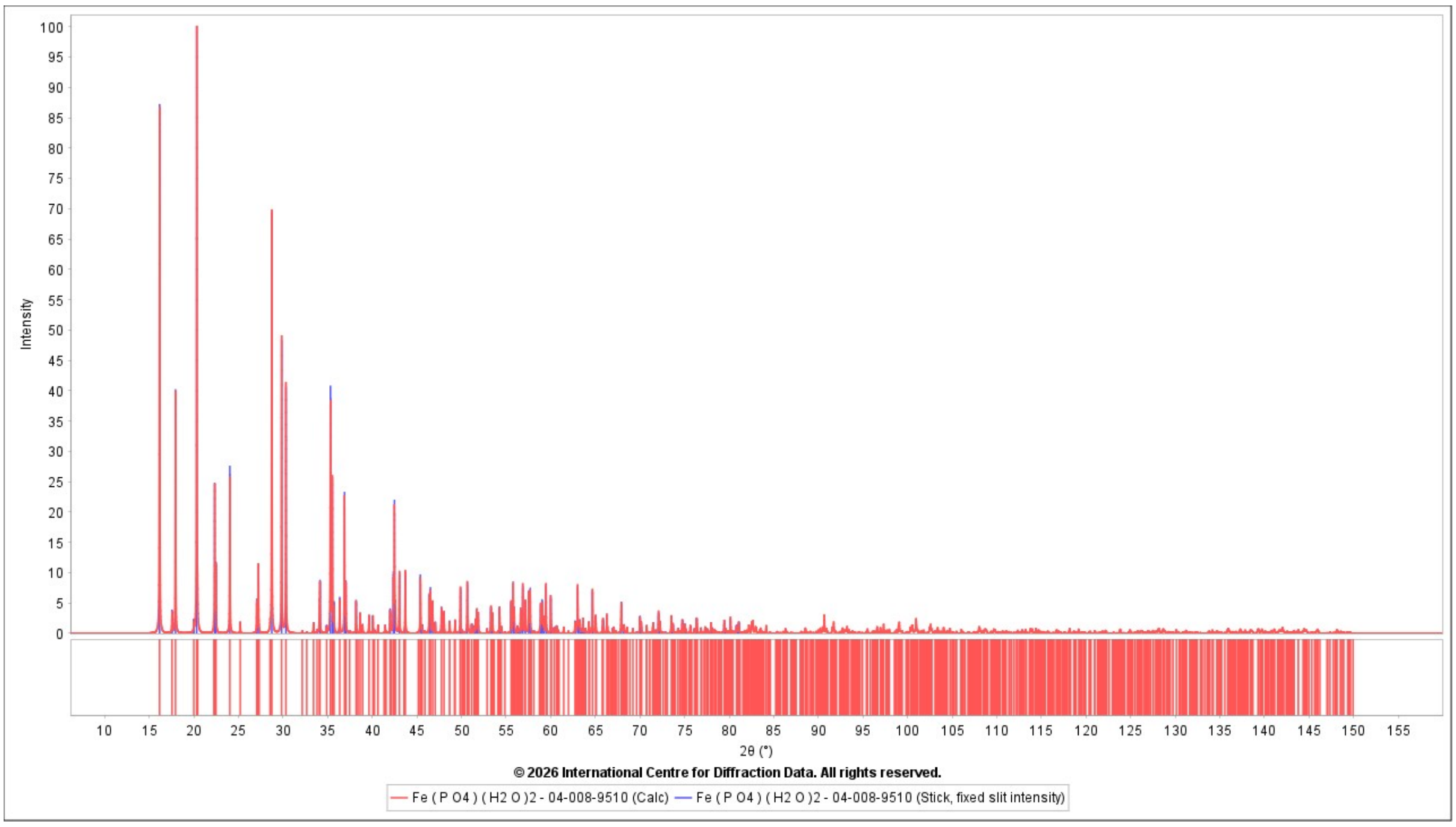

Figure S.5: Diffraction pattern of pure $FePO_4$ $(H_2O)_2$*

Visualization Nernst Equation for Fe-oxidation

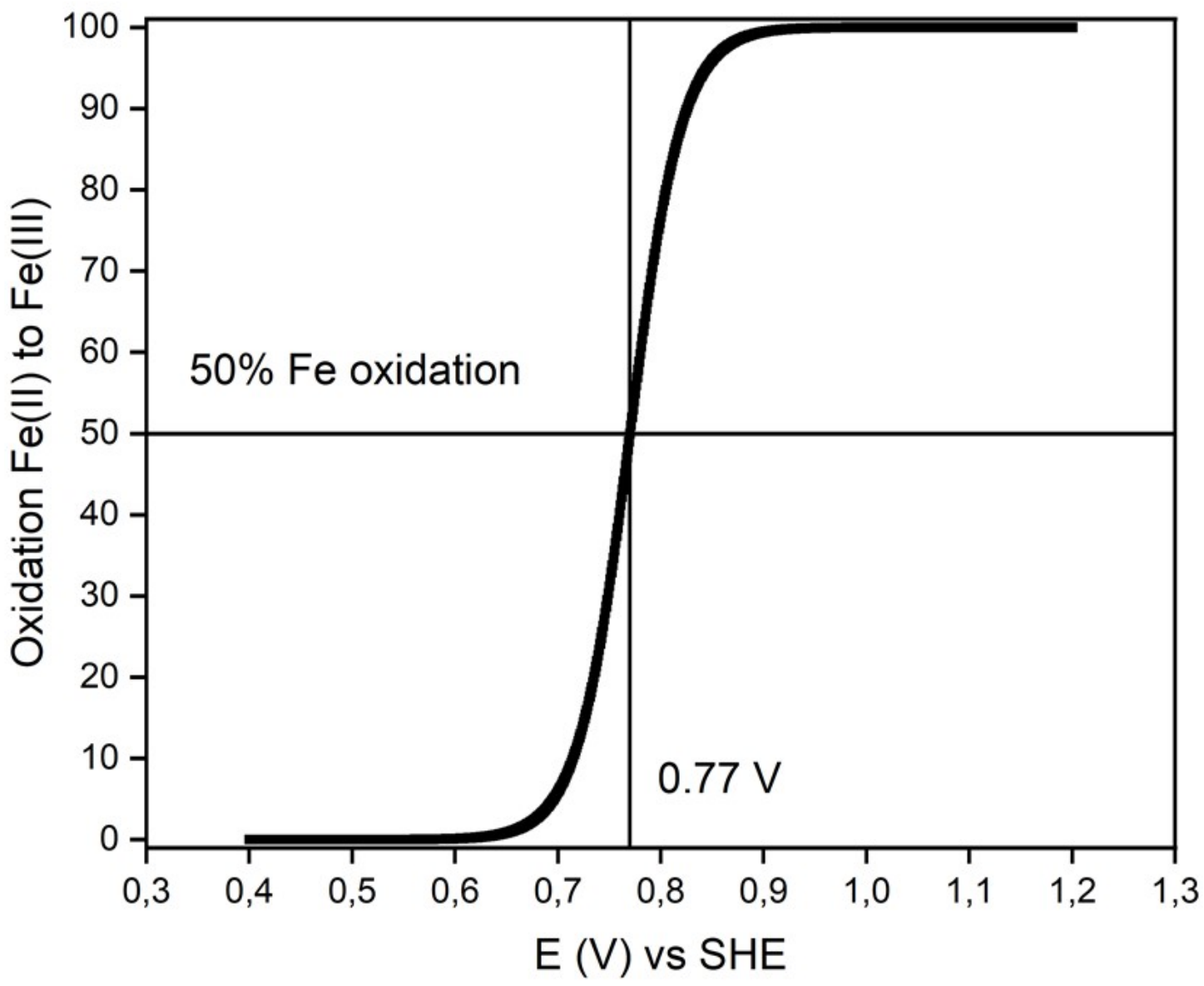

Figure S.6: Dependence of Fe oxidation on the cell potential according to the Nernst Equation. Note that when [Fe^{3+}] = [Fe^{2+}], $E=E^0$.

XRD-analysis Cu deposit

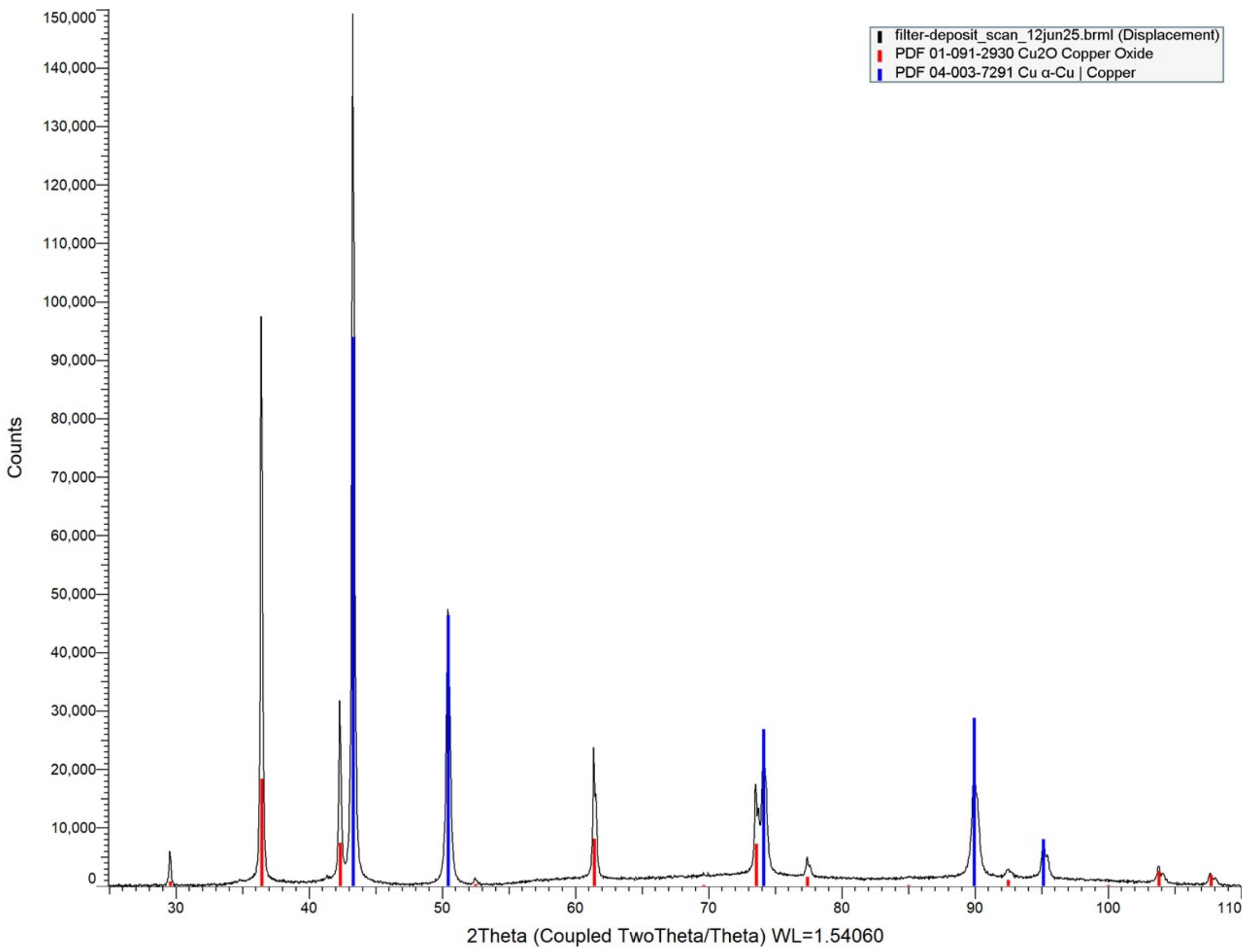

Figure S.7: XRD-diffractogram of the cathode deposit during Fe electro-oxidation

Results Chronopotentiometry During Electrochemical Purification

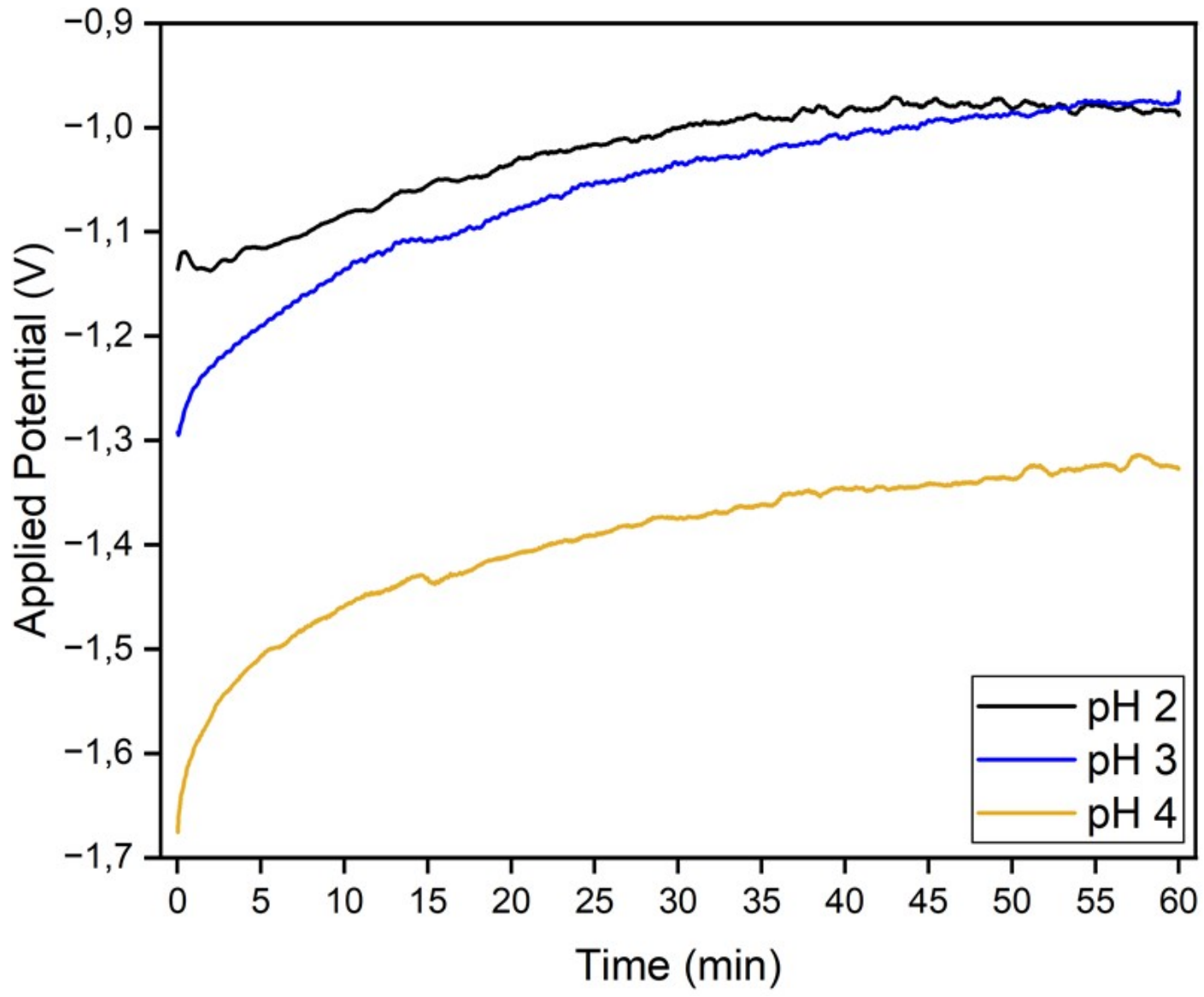

Figure S.8: course of the applied potential during the Cu-deposition process.

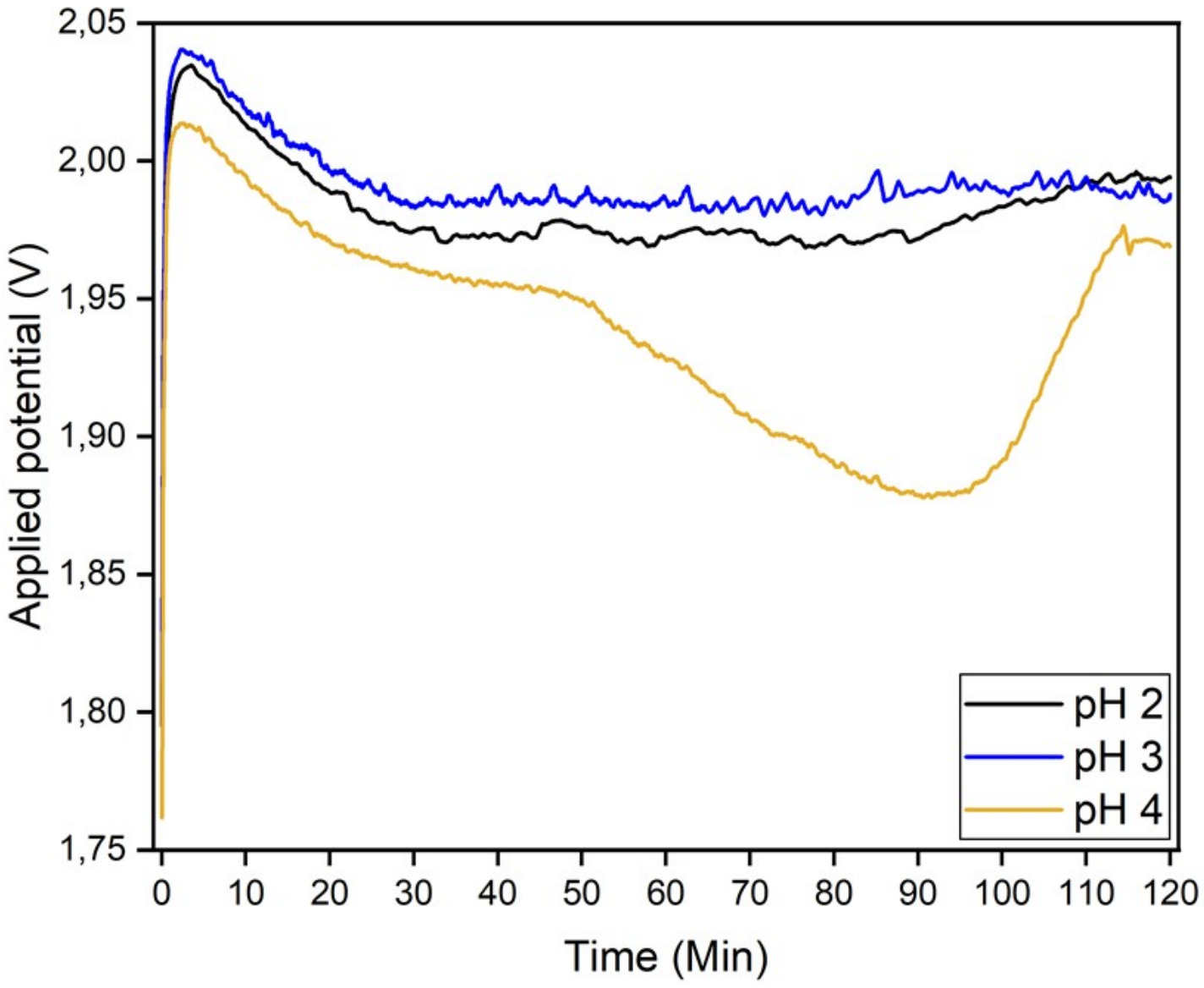

Figure S.9: course of the applied potential during the Fe-oxidation process.
